# Self-nanoemulsifying drug delivery system (SNEDDS) enhances the antidiabetic potential of *Averrhoa bilimbi* L. leaf extract: Integrated LC–MS/MS metabolomics, network pharmacology, and alpha-amylase inhibition

**DOI:** 10.1016/j.toxrep.2026.102259

**Published:** 2026-04-23

**Authors:** Ratih Aryani, Sarah Sarah, Umi Yuniarni, Haura Syabihah, Taufik Muhammad Fakih

**Affiliations:** Department of Pharmacy, Faculty of Mathematics and Natural Sciences, Universitas Islam Bandung, Jl. Ranggagading, Bandung 40116, Indonesia

**Keywords:** *Averrhoa bilimbi* L., LC–MS/MS metabolite profiling, Network pharmacology, Self-nanoemulsifying drug delivery system (SNEDDS), Antidiabetic activity

## Abstract

*Averrhoa bilimbi* L. has long been utilized in traditional medicine and is increasingly investigated for its metabolic benefits, particularly in diabetes management. However, the molecular mechanisms underlying its antidiabetic activity and the influence of formulation strategies on its efficacy remain insufficiently understood. This study aimed to elucidate the antidiabetic potential of *Averrhoa bilimbi* L. leaf metabolites through an integrated approach combining LC–MS/MS profiling, network pharmacology analysis, and formulation into a self-nanoemulsifying drug delivery system (SNEDDS). Metabolites were identified using LC–MS/MS and subjected to in silico target prediction, followed by the collection of Type 2 Diabetes Mellitus-related genes to determine overlapping targets. Protein–protein interaction (PPI) analysis and maximal clique centrality (MCC) were employed to identify key hub proteins. Network pharmacology analysis revealed 434 shared targets, highlighting the involvement of the PI3K–AKT signaling pathway as a central mechanism. The optimized SNEDDS formulation demonstrated high transmittance (81.5%), nanoscale droplet size (157.7 ± 4.90 nm), rapid emulsification time (57 s), and satisfactory stability. In vitro α-amylase inhibition assays showed a significant reduction in IC₅₀ values from 440.92 ± 10.96 µg/mL in the crude extract to 3.75 ± 0.09 µg/mL in the SNEDDS formulation. Nevertheless, the observed enhancement should be interpreted cautiously due to the inherent inhibitory activity of the blank SNEDDS system. In general, *Averrhoa bilimbi* L. exhibits promising antidiabetic activity through multitarget modulation, although further experimental validation is necessary to confirm these findings.

## 1 Introduction

Indonesia is one of the countries with the highest levels of plant biodiversity in the world. Numerous plant species with potential medicinal value grow abundantly across various regions of the country [1], [2]. This condition provides substantial opportunities for the exploration and development of natural product–based medicines. The utilization of medicinal plants is supported not only by traditional knowledge and empirical use. Increasing scientific evidence has also validated their pharmacological potential. Advances in pharmaceutical science enable systematic evaluation of herbal resources [3]. One tropical plant that grows widely in Indonesia is *Averrhoa bilimbi* L. This plant is commonly known as bilimbi or cucumber tree. It is distributed throughout many regions due to favorable climatic conditions [4]. Historically, the plant is believed to have originated from the Maluku Islands. It subsequently spread across Indonesia through cultivation and natural adaptation [5]. Among its various parts, the leaves are commonly used in traditional medicine. The leaves are particularly valued because of their bioactive properties. Phytochemical investigations have demonstrated that the leaves contain diverse secondary metabolites [6]. These metabolites include alkaloids, flavonoids, tannins, polyphenols, and saponins. Such compounds are known to exhibit diverse biological activities. The coexistence of multiple metabolites suggests synergistic pharmacological effects. Multicomponent herbal extracts are particularly relevant for metabolic disorders [7], [8]. Their multitarget nature may enhance therapeutic efficacy. Therefore, *Averrhoa bilimbi* L. leaf extract is considered a promising natural antidiabetic candidate.

Type 2 Diabetes Mellitus is an endocrine metabolic disorder characterized by chronic hyperglycemic conditions. The disease results from insufficient insulin production, pancreatic β-cell dysfunction, or reduced cellular response to insulin. Insulin resistance plays a major role in disease progression [9], [10]. Type 2 Diabetes has become a serious global health problem. According to recent global estimates, approximately 589 million adults aged 20–79 years are currently living with diabetes worldwide [11]. This number corresponds to nearly one in nine adults globally. The prevalence of diabetes continues to increase at an alarming rate. Projections indicate that the global diabetic population may reach approximately 853 million by the year 2050 [12]. This increase is equivalent to nearly one in eight adults worldwide. The rapid rise in prevalence poses significant challenges to healthcare systems. Diabetes is associated with substantial morbidity and mortality. Chronic hyperglycemia leads to progressive metabolic dysregulation. Long-term complications affect multiple organ systems [13], [14]. Cardiovascular disease is one of the most common outcomes. Renal, neural, and retinal complications are also frequently observed. These complications significantly reduce patient quality of life [15]. Effective glycemic control is essential to prevent disease progression. Current therapeutic strategies are not always sufficient. Long-term pharmacotherapy may be associated with adverse effects [16]. Thus, alternative and complementary antidiabetic approaches are urgently needed.

Secondary metabolites play a crucial role in the antidiabetic activity of *Averrhoa bilimbi* L. leaf extract. Alkaloids, flavonoids, tannins, polyphenols, and saponins contribute to antihyperglycemic effects through multiple mechanisms. One important mechanism involves inhibition of α-glucosidase and α-amylase enzymes [17], [18]. These enzymes are responsible for carbohydrate digestion in the gastrointestinal tract. Their inhibition reduces glucose release after meals. As a result, postprandial blood glucose levels can be suppressed. Certain metabolites also inhibit dipeptidyl peptidase-4 activity. DPP-4 inhibition prevents degradation of incretin hormones [19]. Increased incretin levels enhance insulin secretion. Phytochemicals may also influence intestinal glucose transporters. Modulation of SGLT1 and GLUT2 reduces glucose absorption in the intestine [20]. Additional mechanisms involve regulation of insulin signaling pathways. Inhibition of protein tyrosine phosphatase 1B enhances insulin sensitivity [21]. Activation of PI3K and AMPK pathways improves glucose uptake. Other signaling pathways such as p38 MAPK and PPAR-γ are also involved [22]. Increased GLUT4 translocation facilitates glucose entry into peripheral tissues. These mechanisms reflect the multitarget nature of the extract [23]. Synergistic interactions among metabolites may amplify therapeutic effects. Such complexity cannot be explained by single-target approaches. Comprehensive mechanistic investigation is therefore required.

Several experimental studies have demonstrated the antihyperglycemic activity of *Averrhoa bilimbi* L. leaf extract. Ethanolic leaf extracts have shown strong inhibitory activity against carbohydrate-digesting enzymes [24]. *In vitro* studies reported α-amylase inhibition comparable to standard antidiabetic drugs. *In vivo* investigations further confirmed glucose-lowering effects in diabetic animal models [25], [26]. Administration of ethanolic extracts resulted in significant reductions in fasting blood glucose levels. Dose-dependent antihyperglycemic effects were consistently observed [27], [28]. Some studies also reported protective effects on pancreatic tissue. Improvement of pancreatic β-cell integrity was noted following extract administration. Regeneration of damaged pancreatic cells has been suggested in experimental models [29], [30]. In addition to antihyperglycemic activity, the extract exhibits strong antioxidant properties. High free radical scavenging activity has been reported. Oxidative stress plays a critical role in diabetes progression. Chronic hyperglycemia induces excessive production of reactive oxygen species [31], [32]. Accumulation of free radicals causes irreversible cellular damage. Endogenous antioxidant defenses are often insufficient in diabetic conditions. Exogenous antioxidants can help suppress oxidative stress [33], [34]. The antioxidant activity of the extract supports its antidiabetic efficacy. Dual antihyperglycemic and antioxidant actions are therapeutically advantageous. These properties strengthen the potential of the extract as a functional antidiabetic agent. However, formulation challenges remain a major limitation.

Despite promising biological activity, development of herbal extracts into effective oral formulations remains challenging. Many phytochemicals exhibit poor aqueous solubility. Limited solubility reduces dissolution in gastrointestinal fluids [35]. Hence, oral bioavailability is often suboptimal. Chemical instability further compromises therapeutic effectiveness. These limitations hinder clinical translation of herbal medicines. Self-nanoemulsifying drug delivery systems offer an effective formulation strategy. SNEDDS are isotropic mixtures of oils, surfactants, and cosurfactants. They spontaneously form nanoemulsions upon contact with gastrointestinal fluids [36]. Nanoemulsion formation increases surface area for absorption. Enhanced solubilization improves oral bioavailability of phytochemicals. SNEDDS also improve formulation stability and reproducibility. Integration of SNEDDS is particularly suitable for complex herbal extracts. On the other hand, formulation improvement alone does not explain therapeutic mechanisms. Advanced analytical approaches are therefore required. LC–MS/MS enables comprehensive metabolite profiling of herbal extracts [37]. Network pharmacology reveals multitarget interactions at the systems level. This integrated strategy provides a comprehensive framework for antidiabetic drug development.

## 2 Materials and methods

### 2.1 Materials

The materials used in this study included fresh leaves of *Averrhoa bilimbi* L., obtained from the Indonesian Spice and Medicinal Crops Research Institute (IP2SI) Manoko, Lembang District, West Bandung Regency, Indonesia. Botanical identification of the plant material was conducted at Herbarium Bandungense, School of Life Sciences and Technology, Institut Teknologi Bandung, and authenticated under certificate number 1226/IT1.C11.2/TA.00/2025. Acarbose tablets (OGB Dexa) were used as a reference antidiabetic drug. Ethanol (70%) was used as the extraction solvent. Propylene glycol, Tween 20, and Tween 80 (Merck) were used as formulation components. Virgin coconut oil (VCO) was obtained from Brataco and used as the oil phase in SNEDDS formulation. For LC–MS/MS analysis, LC–MS grade solvents including methanol, acetonitrile, and water were used. Formic acid (LC–MS grade) was employed as a mobile phase modifier. All reagents used for LC–MS/MS analysis were of analytical or LC–MS grade to ensure optimal detection sensitivity. Nylon membrane syringe filters (0.22 µm) were used for sample filtration prior to injection. Standard laboratory reagents and chemicals of analytical grade were obtained from the Pharmaceutical Research Laboratory, Department of Pharmacy, Faculty of Mathematics and Natural Sciences, Universitas Islam Bandung. Distilled water was used throughout the experimental procedures where required. All materials were used without further purification.

### 2.2 Instrumentation

The instrumentation used in this study included a blender, separatory funnel, desiccator, and hotplate (Thermo Scientific Cimarec). A condenser, crucible, magnetic stirrer, maceration vessel, and standard laboratory glassware were employed during extraction and sample preparation. An analytical balance (Ohaus) was used for accurate weighing of materials. An oven (Memmert) was used for drying processes. A vacuum rotary evaporator (Büchi Rotavapor R-3) was employed for solvent removal under reduced pressure. A water bath was used for further solvent evaporation and temperature-controlled processes. Sample homogenization was performed using a vortex mixer (Thermo Scientific Maxi Mix II). Sonication was carried out using a sonicator (Branson 2800). Particle size distribution and polydispersity index were determined using a Particle Size Analyzer (Horiba SZ-100). pH measurements were performed using a pH meter (Ohaus). Absorbance measurements were conducted using a UV–Visible spectrophotometer (Shimadzu UV-1800). Centrifugation was performed using a centrifuge (Benchmark LC-8 Series). High-temperature treatments were carried out using a muffle furnace (Ovan). LC–MS/MS analysis was performed using an ultra-high-performance liquid chromatography system coupled with a triple quadrupole mass spectrometer (UHPLC–MS/MS). Chromatographic separation was achieved using a reversed-phase C_18_ analytical column. The mass spectrometer was equipped with an electrospray ionization (ESI) source operating in both positive and negative ion modes. Data acquisition and processing were conducted using LabSolutions software (version 5.99, Shimadzu Corporation, Kyoto, Japan). Sample filtration prior to LC–MS/MS injection was performed using syringe filters. All instrumentation was operated according to standard operating procedures.

### 2.3 Preparation of *Averrhoa bilimbi* L. leaf ethanolic extract

The ethanolic extract of *Averrhoa bilimbi* L. leaves was prepared using the maceration method. Dried leaf simplicia were initially pulverized using a blender to obtain a fine and uniform powder. The powdered material was sieved to ensure particle size homogeneity. The dry powder was accurately weighed prior to extraction. Maceration was carried out using 70% ethanol as the extraction solvent. A solvent-to-material ratio of 1:6 (w/v) was applied during the first maceration step. The mixture was placed in a tightly closed container. The maceration process was conducted at room temperature. The extraction was allowed to proceed for 24 h. Occasional stirring was performed to improve solvent penetration and extraction efficiency. After completion of maceration, the mixture was filtered to obtain the first filtrate. The remaining solid residue was collected for further extraction. Remaceration was performed twice using 70% ethanol at a ratio of 1:3 (w/v). Each remaceration step was conducted for 24 h under identical conditions. The filtrates obtained from all maceration steps were combined into a single solution. The combined filtrate was concentrated under reduced pressure using a vacuum rotary evaporator. Concentration was continued until most of the solvent was removed. Further solvent evaporation was performed using a water bath. A viscous ethanolic extract was obtained at the end of the process. The extraction yield was calculated as the ratio of the weight of the concentrated extract to the initial weight of the dried leaf powder.

### 2.4 Qualitative phytochemical profiling of the ethanolic leaf extract

Phytochemical screening was conducted to qualitatively identify secondary metabolites present in the ethanolic leaf extract. A total of 1 g of the concentrated extract was accurately weighed for analysis. The extract was dissolved in 100 mL of 70% ethanol to prepare a test solution. The solution was homogenized thoroughly before testing. Qualitative phytochemical tests were performed following standard laboratory procedures. Alkaloids were identified using specific precipitation reactions with appropriate reagents. Flavonoids were detected through characteristic color reaction assays. Polyphenols were identified based on complex formation reactions. Tannins were examined using colorimetric reactions with specific reagents. Saponins were detected using the foam formation test. The presence of stable foam indicated a positive result for saponins. Anthraquinones were identified using alkaline reagent reactions. Monoterpenes were examined through characteristic color change reactions. Sesquiterpenes were evaluated using similar qualitative reactions. Triterpenoids were identified using specific reagent-based tests. Steroids were detected using Liebermann–Burchard-type reactions. All tests were conducted under controlled laboratory conditions. Observations were made based on the formation of precipitates, foam, or color changes. Each observation was recorded systematically. The results were used to confirm the presence or absence of each class of secondary metabolite.

### 2.5 LC–MS/MS-based metabolite profiling of the ethanolic leaf extract

LC–MS/MS analysis was conducted to comprehensively characterize the metabolite profile of the ethanolic leaf extract of *Averrhoa bilimbi* L. A defined amount of the concentrated extract was accurately weighed and dissolved in LC–MS grade methanol to prepare a stock solution. The solution was vortexed and sonicated for several minutes to ensure complete homogenization and efficient metabolite extraction. The sample was then centrifuged to remove insoluble particles, and the clear supernatant was collected. Prior to analysis, the solution was filtered through a 0.22 µm membrane syringe filter and transferred into LC–MS compatible vials. Chromatographic separation was performed using a reversed-phase C_18_ column under controlled temperature conditions. The mobile phase consisted of LC–MS grade water (solvent A) and acetonitrile (solvent B), both containing 0.1% formic acid to enhance ionization efficiency. A gradient elution program was applied to achieve optimal separation of metabolites with different polarities, while the flow rate and injection volume were kept constant throughout the analysis. Mass spectrometric detection was carried out using an electrospray ionization (ESI) source operated in both positive and negative ionization modes. Full-scan MS spectra were acquired over a wide mass range, followed by data-dependent MS/MS analysis using collision-induced dissociation (CID) to generate diagnostic fragment ions. Metabolite annotation was performed through a multi-step workflow combining accurate mass measurement, MS/MS fragmentation pattern analysis, and spectral matching against publicly available databases, including MassBank, METLIN, and mzCloud. The mass accuracy was evaluated based on mass error (expressed in ppm), with a tolerance threshold of ≤ 5 ppm for tentative identification. Fragmentation patterns of detected ions were compared with reference spectra from databases and relevant literature to confirm structural consistency. Retention behavior was also considered by comparing chromatographic elution order with reported polarity characteristics of candidate compounds. Each metabolite assignment was classified according to confidence levels following the Metabolomics Standards Initiative (MSI) guidelines, where Level 1 indicates confirmed identification using authentic standards, Level 2 represents putatively annotated compounds based on spectral similarity, and Level 3 corresponds to tentative compound classes. In this study, due to the absence of authentic reference standards, the reported metabolites were categorized as Level 2 (putatively annotated compounds).

### 2.6 Visualization of two-dimensional (2D) chemical structures

The two-dimensional (2D) chemical structures of the metabolites identified from *Averrhoa bilimbi* L. leaf extract were generated to support structural analysis and subsequent *in silico* evaluation. Chemical structure information for each metabolite was retrieved from publicly available databases, including PubChem (https://pubchem.ncbi.nlm.nih.gov/) [38]and ChemSpider (https://www.chemspider.com/) [39], based on confirmed compound identities from LC–MS/MS annotation. The 2D structures were drawn and visualized using ChemDraw Professional software (version 16.0, PerkinElmer), which allows standardized depiction of chemical bonds, functional groups, and stereochemical features [40]. Structural validation was performed by cross-checking molecular formulas, exact masses, and functional group consistency with reported literature data. The generated 2D structures were saved in standard formats (PNG and MOL) to ensure compatibility with downstream computational tools. These visual representations were used for qualitative comparison of structural diversity among metabolites. Special attention was given to identifying key pharmacophoric features such as hydroxyl groups, aromatic rings, heterocycles, and alkyl chains. Such features are known to influence solubility, permeability, and biological activity. The 2D visualization step ensured structural clarity prior to pharmacokinetic and drug-likeness evaluation. This approach facilitated systematic analysis of structure–property relationships. Only well-defined and structurally validated compounds were carried forward for ADME prediction.

### 2.7 In silico ADME and drug-likeness prediction using SwissADME

Pharmacokinetic properties and drug-likeness of the identified metabolites were evaluated using the SwissADME web server (http://www.swissadme.ch/) [41]. Canonical SMILES strings for each compound were obtained from PubChem or generated using ChemDraw and subsequently used as input for SwissADME analysis. The platform was employed to predict key absorption, distribution, metabolism, and excretion (ADME) parameters relevant to oral administration. Predicted gastrointestinal (GI) absorption, blood–brain barrier (BBB) permeability, and P-glycoprotein (P-gp) substrate status were recorded. Physicochemical descriptors, including molecular weight, topological polar surface area (TPSA), number of hydrogen bond donors and acceptors, and lipophilicity indices (Log P values), were also calculated. Drug-likeness was assessed according to established criteria, including Lipinski’s Rule of Five, Veber’s rule, Egan’s rule, and Ghose filter. Compounds with no more than one violation of Lipinski’s criteria were considered acceptable. Oral bioavailability was further evaluated using the bioavailability radar provided by SwissADME. Water solubility predictions were recorded using the ESOL model. Compounds predicted to have extremely low solubility were carefully evaluated for formulation relevance. The BOILED-Egg model was used to visualize passive gastrointestinal absorption and brain penetration tendencies. These predictions provided insight into the feasibility of oral delivery. SwissADME analysis enabled early identification of compounds with favorable pharmacokinetic profiles. This screening step reduced the likelihood of advancing compounds with poor ADME properties. The resulting data supported rational prioritization of metabolites for formulation development. Integration of ADME prediction with formulation design enhanced translational relevance. This approach aligns with contemporary strategies in natural product-based drug development.

### 2.8 Prediction of molecular targets associated with *Averrhoa bilimbi* L. leaf metabolites

Target prediction was conducted to identify potential protein targets associated with the bioactive metabolites of *Averrhoa bilimbi* L. leaf extract. Canonical SMILES of the selected metabolites were used as input for target prediction using SwissTargetPrediction (http://www.swisstargetprediction.ch/) with the organism set to *Homo sapiens* [42]. The platform predicts likely molecular targets based on chemical similarity and known ligand–target interactions. Only targets with non-zero probability scores were retained to ensure prediction reliability. Duplicate targets across metabolites were removed to generate a non-redundant target list. The predicted targets were further curated to remove ambiguous or uncharacterized proteins. Identified targets were annotated using UniProt to confirm protein identity and biological relevance [42]. Functional relevance to glucose metabolism, insulin signaling, inflammation, and oxidative stress was assessed through literature screening. Targets involved in metabolic regulation and signal transduction were prioritized. This step enabled systematic mapping of metabolite–target associations. The resulting dataset provided a molecular basis for exploring antidiabetic mechanisms. Predicted targets were subsequently integrated with disease-related targets. This approach supports mechanism-oriented analysis rather than single-target assumptions. Target prediction served as a bridge between chemical profiling and systems-level analysis. The curated target list was used for overlap analysis with Type 2 Diabetes Mellitus–related genes. This process enhanced the biological relevance of subsequent network analyses.

### 2.9 Integration of metabolite targets with type 2 diabetes mellitus

Type 2 Diabetes Mellitus–related genes were collected from GeneCards (https://www.genecards.org/) [43], OMIM (https://www.omim.org/) [44], and the GEO database using the keyword “Type 2 Diabetes Mellitus” (https://www.ncbi.nlm.nih.gov/geo/) [45]. For the GEO database, expression datasets were screened under the restriction of *Homo sapiens* and were included only when they contained both T2DM patient samples and non-diabetic controls, provided raw or processed gene expression data, and represented disease-relevant tissues or cells, such as blood, skeletal muscle, adipose tissue, liver, or pancreatic islets. Datasets lacking an appropriate control group, involving non-human samples, or focusing on unrelated pathological conditions were excluded. From GEO, relevant expression datasets were selected to represent disease-associated transcriptional changes. Differentially expressed genes from the selected GEO datasets were filtered using the thresholds of adjusted p < 0.05 and |log2 fold change| ≥1 to ensure statistical and biological relevance. To improve disease specificity, only genes associated with glucose metabolism, insulin resistance, lipid dysregulation, inflammation, or oxidative stress were retained for integration. Disease-related targets from all sources were merged and duplicates were removed to obtain a comprehensive diabetes gene set. Overlapping targets between *Averrhoa bilimbi* L. metabolite targets and diabetes-associated genes were identified using Venn diagram analysis [46]. These shared targets were considered potential therapeutic nodes mediating antidiabetic effects. The intersecting gene set was imported into the STRING database (version 12.0) for protein–protein interaction (PPI) analysis [47]. The organism was restricted to *Homo sapiens*. A medium confidence interaction score threshold of 0.4 was applied to balance coverage and reliability. Disconnected nodes were excluded from further analysis. The resulting PPI network was exported in TSV format. Network visualization and analysis were performed using Cytoscape software (version 3.10.3) [48]. Topological properties of the network were calculated to evaluate interaction density and connectivity. This network provided insight into the molecular interplay among shared targets. The PPI analysis supported identification of central regulatory proteins. These interactions formed the basis for hub gene screening. The constructed network reflects system-level involvement in diabetes-related signaling.

### 2.10 Gene ontology and pathway-based functional interpretation

Functional enrichment analysis was conducted to elucidate the biological significance of overlapping targets. Gene Ontology (GO) enrichment analysis was performed using the STRING platform to classify targets into biological processes (BP), molecular functions (MF), and cellular components (CC) [49]. Kyoto Encyclopedia of Genes and Genomes (KEGG) pathway analysis was used to identify enriched signaling pathways associated with diabetes pathophysiology [50]. Enrichment significance was evaluated using false discovery rate (FDR)–adjusted *p*-values. Terms with FDR <0.05 were considered statistically significant. The top enriched GO terms and KEGG pathways were selected based on gene count and significance. Visualization of enrichment results was performed using the Bioinformatics online platform (https://www.bioinformatics.com.cn/). Bar plots and bubble charts were generated to illustrate enrichment patterns. Enriched terms were interpreted with particular emphasis on biological categories directly relevant to T2DM pathogenesis, including insulin signaling, glucose metabolism, lipid regulation, inflammation, and oxidative stress, in order to maintain disease-focused functional interpretation. Enriched biological processes related to insulin signaling, glucose metabolism, lipid regulation, inflammation, and oxidative stress were highlighted. KEGG analysis emphasized pathways relevant to metabolic homeostasis. These results provided mechanistic insight into the antidiabetic potential of *Averrhoa bilimbi* L. metabolites. Functional enrichment supported interpretation of network pharmacology findings. The analysis linked molecular targets to disease-relevant pathways. This step strengthened the mechanistic framework of the study. Enrichment analysis guided subsequent hub gene prioritization. The results established biological plausibility for multitarget regulation.

### 2.11 Network-centered identification of key regulatory nodes

Hub gene analysis was performed to identify key regulatory proteins within the PPI network. Topological analysis was conducted using the CytoHubba plugin in Cytoscape [51]. Maximal Clique Centrality (MCC) was selected as the ranking method due to its robustness in identifying essential nodes [52]. Genes with the highest MCC scores were defined as hub genes. These hub genes were analyzed for pathway association using KEGG annotation. Particular attention was given to the PI3K–AKT signaling pathway due to its central role in insulin action and glucose homeostasis. Subnetwork extraction was performed to visualize interactions among PI3K–AKT–associated hub genes. Network density and connectivity were evaluated to assess regulatory importance. Hub genes involved in signal transduction, kinase activity, and adaptor functions were highlighted. The interaction architecture was examined to identify coordinated regulatory patterns. This analysis emphasized pathway-level rather than isolated protein effects. Hub gene interaction mapping provided insight into multitarget modulation mechanisms. The identified hubs represent potential intervention points for antidiabetic therapy. Network topology supported the systems pharmacology concept of the study. Because hub gene prioritization was derived from integrated prediction-based and transcriptomic datasets, the identified regulatory nodes were interpreted as candidate targets associated with T2DM-related signaling rather than experimentally validated mechanistic effectors. These findings guided interpretation of downstream formulation and activity results. The hub analysis established a molecular rationale for therapeutic relevance. This approach integrates network structure with biological function.

### 2.12 Selection of oil phase and screening of surfactant–cosurfactant systems

The selection of suitable lipid excipients was conducted to ensure optimal solubilization of *Averrhoa bilimbi* L. leaf extract and efficient self-emulsification performance. Preliminary solubility studies were performed to evaluate the solubilization capacity of various oils, surfactants, and cosurfactants toward the extract. Candidate oil phases included medium- and long-chain fatty acids commonly used in lipid-based formulations. Surfactants with high hydrophilic–lipophilic balance (HLB) values were prioritized to promote spontaneous nanoemulsion formation. Cosurfactants were selected based on their ability to enhance interfacial flexibility and reduce emulsification time [53]. Solubility assessment was carried out by adding excess extract to each excipient followed by vortexing and equilibrium incubation. Samples were centrifuged, and the supernatant was visually inspected for clarity. The oil phase exhibiting the highest solubilization capacity was selected for further formulation. Surfactant and cosurfactant combinations were screened to identify systems capable of forming clear emulsions upon aqueous dilution. The compatibility of excipients was considered to avoid phase separation. Selected components were pharmaceutically acceptable and orally safe. This screening step ensured rational excipient selection. The results guided the construction of pseudo-ternary systems. Appropriate excipient pairing is critical for SNEDDS stability. This stage laid the foundation for formulation optimization.

### 2.13 Formulation optimization strategy of SNEDDS incorporating *Averrhoa bilimbi* L. leaf extract

SNEDDS formulation optimization was conducted by systematically varying the ratios of oil, surfactant, and cosurfactant. Based on the selected excipients, a series of formulations were prepared using different oil-to-S_mix_ ratios. S_mix_ was prepared by blending surfactant and cosurfactant at predefined ratios. Each formulation was evaluated for emulsification time, clarity, and visual appearance upon dilution with distilled water. Self-emulsification behavior was assessed under gentle agitation to simulate gastrointestinal conditions. Transmittance measurements were performed using UV–Vis spectrophotometer to evaluate optical clarity. Formulations with high transmittance values were considered indicative of efficient nanoemulsion formation. Emulsification time was recorded to assess dispersion efficiency. Formulations were graded based on visual clarity and dispersion behavior. Systems showing rapid emulsification and clear appearance were shortlisted. The extract was incorporated into selected formulations at a fixed concentration. Compatibility of the extract with the SNEDDS system was assessed. Formulations exhibiting precipitation or turbidity were excluded. Optimization focused on balancing performance and compositional simplicity. The best-performing formulation was selected for further characterization [54]. This approach ensured reproducibility and robustness. Optimized composition was used for subsequent evaluations.

### 2.14 Physicochemical characterization and stability evaluation of the optimized SNEDDS formulation

The optimized SNEDDS formulation was subjected to comprehensive physicochemical characterization. Optical clarity was evaluated by measuring percentage transmittance after dilution with distilled water. Droplet size and polydispersity index were determined using dynamic light scattering. These parameters were used to assess nanoemulsion uniformity and dispersion quality. Emulsification time was measured to evaluate the rapidity of self-emulsification. Dispersibility testing was performed to classify the formulation based on visual grading criteria. Robustness to dilution was assessed in different aqueous media. Stability in acidic and neutral pH conditions was evaluated using 0.1 N HCl and phosphate buffer pH 6.8. Thermodynamic stability was assessed through centrifugation testing. Heating–cooling cycles were conducted to evaluate temperature-induced instability. Freeze–thaw cycles were used to assess resistance to phase separation. Visual inspection was performed after each stress condition. Absence of creaming or separation indicated good stability. These tests confirmed formulation robustness. Physicochemical performance reflects delivery efficiency. Stable nanoemulsions are essential for oral application. This characterization ensured formulation suitability [55].

### 2.15 In vitro evaluation of antidiabetic activity of the optimized SNEDDS formulation

The antidiabetic potential of the optimized SNEDDS formulation was evaluated using an *in vitro* α-amylase inhibition assay. The assay was performed to assess the ability of the formulation to inhibit carbohydrate-hydrolyzing enzymes. The α-amylase solution was prepared in phosphate buffer at pH 7.4. The SNEDDS formulation containing *Averrhoa bilimbi* L. extract was incubated with the enzyme prior to substrate addition. Soluble starch was used as the enzymatic substrate. The reaction mixture was incubated at controlled temperature. Enzymatic activity was terminated using a color-developing reagent. Absorbance was measured spectrophotometrically to quantify enzyme inhibition. Acarbose was used as a positive control. Percentage inhibition was calculated relative to the control. Concentration-dependent inhibition profiles were generated. The assay was performed in triplicate to ensure reproducibility. Results were expressed as mean ± standard deviation. Enhanced inhibition by SNEDDS compared with crude extract was evaluated. Improved activity was attributed to enhanced solubilization. This assay provides functional validation of antidiabetic potential. The results link formulation performance to biological efficacy [56].

## 3 Results

### 3.1 Qualitative phytochemical profile of *Averrhoa bilimbi* L. leaf simplicia and ethanolic extract

The qualitative phytochemical screening demonstrates that *Averrhoa bilimbi* L. leaf simplicia and its ethanolic extract contain a wide range of secondary metabolites, indicating a chemically diverse plant material. As shown in Table 1, alkaloids were consistently detected in both samples using Dragendorff’s, Mayer’s, and Wagner’s reagents. The presence of alkaloids suggests nitrogen-containing compounds that often exhibit strong biological activity even at low concentrations. Polyphenols were positively identified, confirming the abundance of hydroxylated aromatic compounds within the leaves. Flavonoids were detected through the Shinoda test, supporting the presence of conjugated phenolic structures. These compounds are widely recognized for their antioxidant and enzyme-modulating properties. Tannins were also identified via the ferric chloride reaction, indicating polyphenolic compounds capable of interacting with proteins. Such interactions may influence enzyme activity and cellular signaling pathways. Saponins were detected using the froth test, suggesting amphiphilic molecules that can affect membrane permeability. The presence of saponins may enhance the biological accessibility of other coexisting metabolites. Anthraquinones were identified through the Borntrager’s test, indicating the presence of quinone-type compounds. These compounds are often associated with redox activity and cellular stress modulation. In contrast, mono- and sesquiterpenes were not detected in either sample. This absence indicates that volatile terpenoid constituents are not dominant in the leaves. Steroids were clearly detected using the Liebermann–Burchard reaction. Their presence suggests potential interaction with membrane-associated targets. Triterpenoids were also identified, highlighting the abundance of higher molecular weight terpenoid structures.

**Table 1:** Qualitative Phytochemical Screening Results of *Averrhoa bilimbi* L. Leaf Simplicia and Ethanolic Extract.

**Phytochemical Group**	**Test or Reagent**	Results*	
		**Simplicia**	**Extract**
Alkaloid	Dragendorff’s, Mayer, and Wagner	+	+
Polyphenols	Ferric chloride	+	+
Flavonoids	Shinoda test	+	+
Tannis	Ferric chloride	+	+
Saponins	Froth test	+	+
Anthraquinones	Borntrager’s test	+	+
Mono and Sesquiterpenes	Salkowski	-	-
Steroids	Liebermann–Burchard	+	+
Triterpenoids	Liebermann–Burchard	+	+

* Results are expressed as: (-) not detected, (+) detected

The similarity of phytochemical profiles between the simplicia and ethanolic extract indicates that the extraction process did not markedly alter metabolite composition. This observation suggests that 70% ethanol is an effective solvent for extracting both polar and semi-polar compounds. Phenolic compounds such as flavonoids and tannins are known to dissolve well in hydroethanolic systems. Alkaloids also exhibit favorable solubility in ethanol-containing solvents. The dominance of phenolic compounds provides a chemical basis for the reported antioxidant activity of *Averrhoa bilimbi* L. leaves. Antioxidant compounds play an important role in mitigating oxidative stress-related cellular damage. Oxidative stress is closely associated with metabolic and inflammatory disorders. The presence of alkaloids may further contribute to pharmacological effects through enzyme inhibition or receptor modulation. Saponins may act synergistically by enhancing compound permeability across biological membranes. The detection of triterpenoids and steroids supports potential anti-inflammatory and cytoprotective activities. These lipophilic compounds are often associated with modulation of membrane integrity and signaling cascades. The absence of mono- and sesquiterpenes suggests limited contribution from volatile constituents. This indicates that the biological activity of the leaves is primarily driven by non-volatile secondary metabolites. The combined presence of multiple bioactive classes suggests a multifaceted pharmacological potential. Such compositional diversity is characteristic of medicinal plants with broad therapeutic applications. These findings justify further quantitative phytochemical analysis. They also support subsequent bioactivity-guided and mechanistic investigations.

### 3.2 LC–MS/MS-based metabolite profiling of *Averrhoa bilimbi* L. leaf ethanolic extract

**Fig. 1:**
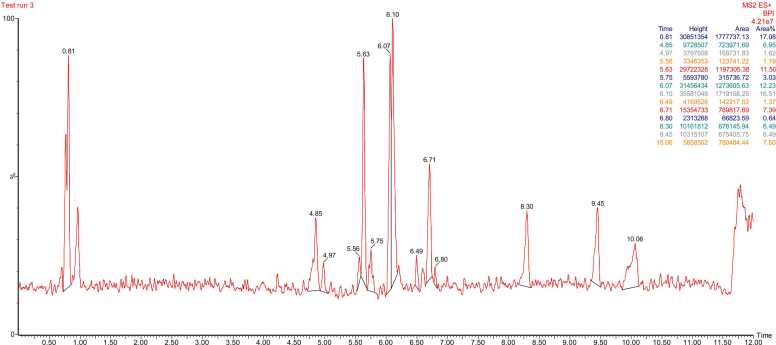
LC–MS/MS Chromatographic Profile of *Averrhoa bilimbi* L. Leaf Ethanolic Extract in Positive Ionization Mode.

The LC–MS/MS profile of the *Averrhoa bilimbi* L. leaf ethanolic extract indicates a broad chemical space that includes both highly polar and strongly lipophilic constituents. The chromatographic trace displayed in Fig. 1 suggests that metabolite signals are distributed across early, mid, and late retention windows, reflecting effective separation under the applied conditions. The retention time span inferred from the identified peaks ranges from 0.81 min to 10.06 min, supporting the presence of compounds with markedly different polarity. The earliest dominant peak corresponds to Mol1 at 0.81 min (*m/z* 217.1422), which also shows the highest relative abundance at 17.08%. Such early elution is consistent with a polar, oxygen-rich carboxylic acid scaffold that ionizes efficiently in positive mode. A cluster of prominent peaks appears around 6 min, indicating enrichment of moderately polar nitrogen-containing metabolites. In this mid-retention region, Mol7 at 6.07 min reaches 12.23% abundance and Mol8 at 6.10 min reaches 16.51% abundance, showing that the extract is strongly dominated by heterocycle-associated features in the central chromatographic window. The appearance of Mol6 at 5.75 min (*m/z* 364.1876) further supports the presence of conjugated aromatic systems that typically elute later than small polar acids. A clear transition toward more hydrophobic chemistry occurs after 6.7 min, where a carotenoid-like compound is detected at 6.71 min with *m/z* 537.4444. Lipid-like and long-chain aromatic metabolites become more evident at later times, including peaks at 8.30 min and 9.45 min that each contribute 6.49% abundance. The late-eluting Mol14 at 10.06 min (*m/z* 436.3800) indicates the presence of a large amphiphilic structure with both heteroatoms and an extended hydrocarbon chain. The coexistence of early polar peaks and late lipophilic peaks suggests that 70% ethanol extracted metabolites across a wide polarity spectrum. The dominance of multiple intense peaks rather than a single compound implies that bioactivity may arise from several constituents acting in parallel. The mid-retention enrichment of N-containing metabolites is particularly relevant because such scaffolds frequently interact with enzymes and signaling proteins. The chemical heterogeneity captured by the chromatogram provides a robust basis for compound-level annotation and downstream mechanistic interpretation.

A detailed view of the annotated metabolites shows that the extract contains fourteen compounds with distinct molecular formulas, mass features, and abundance contributions, spanning acids, phenolics, alkaloid-like structures, lipid-related molecules, and a carotenoid. This diversity is summarized in Table 2, which also shows that the profile is dominated by a small subset of high-abundance metabolites rather than evenly distributed signals across all identified compounds. Mol1 is the most abundant component (17.08%) and is assigned the formula C_11_H_20_O_4_, supporting a polyhydroxylated cyclohexane carboxylic acid framework with high polarity and early elution. Mol8 is the second most abundant metabolite (16.51%) with formula C_18_H_21_NO_2_, indicating a nitrogen-containing heterocycle with moderate polarity and strong chromatographic representation. Mol7 also contributes substantially (12.23%) and carries formula C_18_H_21_NO_5_, suggesting additional oxygenation that can increase hydrogen-bonding potential and modulate bioactivity. A terpene-like acid is represented by Mol5 (11.5%; C_20_H_32_O_2_), which introduces a hydrophobic skeleton with a terminal carboxyl group that may support membrane-associated interactions while retaining some polarity. The carotenoid fraction is captured by β-carotene (Mol10) at 7.39% with formula C_40_H_56_, providing a plausible antioxidant reservoir due to its extended conjugated system. Two long-chain phenolic derivatives, Mol12 and Mol13, each contribute 6.49% and have formulas C_25_H_42_O_3_ and C_25_H_44_O_3_, indicating strong lipophilicity combined with phenolic functionality. A phenethylamide-like lipid derivative is represented by Mol9 (1.37%; C_23_H_37_NO_2_), which may reflect membrane-active or transport-relevant chemistry. The presence of a sphingoid base-like molecule (Mol4; 1.19%; C_18_H_37_NO_3_) supports the occurrence of lipid signaling–related scaffolds in the extract. Lower-abundance aromatic metabolites such as Mol3 (1.62%; C_20_H_18_O_5_) and Mol6 (3.03%; C_23_H_25_NO_3_) contribute chemically informative scaffolds despite smaller percentages. The steroid-like structure Mol11 appears at very low abundance (0.64%; C_25_H_42_O_2_), indicating that sterol-type components are present but not dominant. Importantly, the combined dominance of Mol1, Mol8, Mol7, and Mol5 accounts for a large fraction of the profile, emphasizing that a few metabolites likely drive major chemical contributions. This composition pattern supports a multitarget phytochemical system in which polar acids, heterocycles, and lipophilic antioxidants can contribute complementary biological roles.

**Table 2:** LC–MS/MS-Based Metabolite Identification of *Averrhoa bilimbi* L. Leaf Ethanolic Extract.

**No**	**Retention Time (min)**	**Observed*****m/z***	**Molecular Weight (Da)**	**Mass Error (ppm)**	**Molecular Formula**	**Relative Abundance (%)**	**Prediction Compound Name**	**Compound ID**	**Canonical SMILES**	**Putative Annotation**	**Key MS/MS Fragments (*****m/z*****)**	**Library Match Score (%)**	**MSI Level**
1	0.81	217.1422	216.1	≤ 5	C_11_H_20_O_4_	17.08	(1 R,2S,3 R,4 R,5 R)-3,4-dihydroxy-5-methyl-2-(propan-2-yl)cyclohexane-1-carboxylic acid	Mol1	O[C@@H]1[C@H]([C@@H](C[C@H]([C@H]1 O)C)C(=O)O)C(C)C	Cyclohexanecarboxylic acid derivative	199, 171, 153	82	Level 2
2	4.85	274.1409	273.1	≤ 5	C_16_H_19_NO_3_	6.95	4'-o-methylnorbelladine	Mol2	COC1 =C(C=C(C=C1)CNCCC2 =CC=C(C=C2)O)O	4′-O-methylnorbelladine	256, 238, 121	88	Level 2
3	4.97	339.1221	338.1	≤ 5	C_20_H_18_O_5_	1.62	Psoralenol	Mol3	CC1(C(CC2 =C(O1)C=CC(=C2)C3 =COC4 =C(C3 =O)C=CC(=C4)O)O)C	Psoralenol	321, 293, 265	85	Level 2
4	5.56	316.2825	315.3	≤ 5	C_18_H_37_NO_3_	1.19	4-Hydroxy-8-sphingenine	Mol4	CCCCCCCCC/C=C/CCC[C@H]([C@H]([C@H](CO)N)O)O	4-Hydroxy-8-sphingenine	298, 280, 262	80	Level 2
5	5.63	305.2401	304.2	≤ 5	C_20_H_32_O_2_	11.5	(3S)-5-[(1S,4aR,8aR)-5,5,8a-trimethyl-2-methylidene-3,4,4a,5,6,7,8,8a-octahydronaphthalen-1-yl]-3-methylpentanoic acid	Mol5	C[C@@H](CC[C@H]1 C(=C)C=C[C@H]2[C@@]1(CCCC2(C)C)C)CC(=O)O	Terpenoid derivative	287, 269, 251	83	Level 2
6	5.75	364.1876	363.2	≤ 5	C_23_H_25_NO_3_	3.03	8-[(2*E*)-3,7-dimethylocta-2,6-dien-1-yl]-7-hydroxy-3-methyl-9H-carbazole-1,4-dione	Mol6	CC(C)=CCC/C(C)=C/Cc1c(O)ccc2c3c([nH]c12)C(=O)C=C(C)C3 =O	Carbazole derivative	346, 318, 290	81	Level 2
7	6.07	332.1500	331.1	≤ 5	C_18_H_21_NO_5_	12.23	Methyl 7-hydroxy-3-methoxy-1-methyl-8-(3-methylbut-2-en-1-yl)-4-oxo-1,4-dihydroquinoline-2-carboxylate	Mol7	COC(=O)c1c(OC)c(=O)c2ccc(O)c(CC=C(C)C)c2n1C	Quinoline derivative	314, 286, 258	86	Level 2
8	6.10	284.1601	283.2	≤ 5	C_18_H_21_NO_2_	16.51	2-[(4E,6E)-3-hydroxynona-4,6-dien-1-yl]-1H-quinolin-4-one	Mol8	CC/C=C/C=C/C(CCC1 =CC(=O)C2 =CC=CC=C2N1)O	Quinolinone derivative	266, 248, 220	84	Level 2
9	6.49	360.2900	359.3	≤ 5	C_23_H_37_NO_2_	1.37	(4E,7S)-7-methoxy-N-(2-phenylethyl)tetradec-4-enimidic acid	Mol9	CCCCCCC[C@@H](C/C=C/CCC(=O)NCCC1 =CC=CC=C1)OC	Long-chain amide derivative	342, 324, 296	79	Level 2
10	6.71	537.4444	536.9	≤ 5	C_40_H_56_	7.39	beta-carotene	Mol10	CC1 =C(C(CCC1)(C)C)/C=C/C(=C/C=C/C(=C/C=C/C=C(/C=C/C=C(/C=C/C2 =C(CCCC2(C)C)C)\C)\C)/C)/C	β-Carotene	444, 119, 69	92	Level 2
11	6.80	375.3266	374.6	≤ 5	C_25_H_42_O_2_	0.64	(1 R,3aR,5aR,7S,9aS,11aR)-1-[(2S)-1-hydroxypropan-2-yl]-3a,6,6,9a,11a-pentamethyl-icosahydro-1H-cyclopenta[*a*]phenanthren-7-ol	Mol11	C[C@@]1(CC[C@@]23 C[C@@H]1 C[C@@H]2CC[C@@H]4[C@@]3(C[C@H](CC4(C)C)O)C)O	Steroid-like compound	357, 339, 321	78	Level 2
12	8.30	391.3221	390.3	≤ 5	C_25_H_42_O_3_	6.49	1-(3,5-dihydroxyphenyl)nonadecan-2-one	Mol12	CCCCCCCCCCCCCCCCCC(=O)CC1 =CC(=CC(=C1)O)O	Phenolic ketone derivative	373, 355, 327	83	Level 2
13	9.45	393.3370	392.3	≤ 5	C_25_H_44_O_3_	6.49	4-heptadecyl-2,3-dimethoxyphenol	Mol13	CCCCCCCCCCCCCCCCCC1 =C(C(=CC(=C1)O)OC)OC	Methoxyphenol derivative	375, 357, 329	82	Level 2
14	10.06	436.3800	435.4	≤ 5	C_27_H_49_NO_3_	7.5	22-hydroxy-1-[(3 R)-3-hydroxypiperidin-1-yl]docosa-2,4-dien-1-one	Mol14	C1C[C@H](CN(C1)C(=O)C=CC=CCCCCCCCCCCCCCCCCCO)O	Long-chain amide derivative	418, 400, 372	80	Level 2

**Fig. 2:**
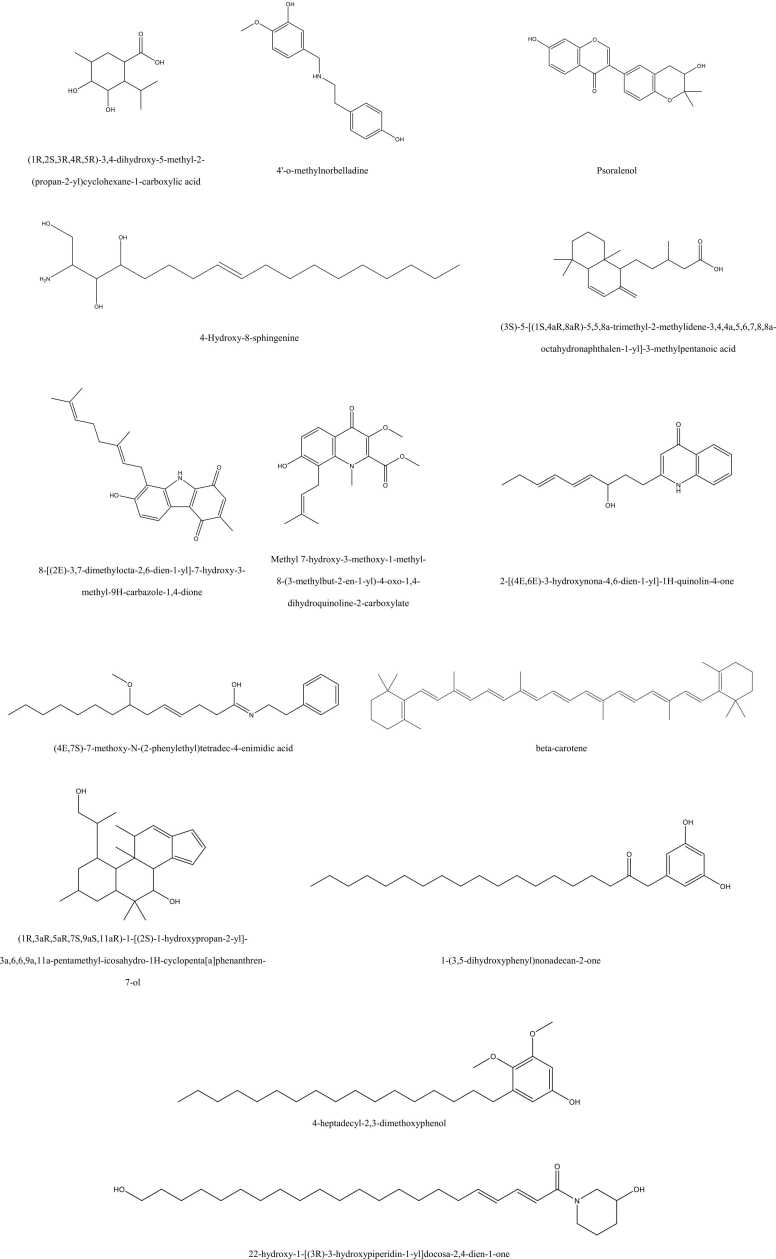
Chemical Structures of Representative Metabolites Identified in *Averrhoa bilimbi* L. Leaf Ethanolic Extract.

Structural depiction of representative compounds provides important mechanistic cues because functional groups and scaffold geometry determine how metabolites may interact with enzymes, receptors, or redox systems. The chemical diversity summarized in Fig. 2 highlights that the extract is not restricted to one scaffold family but instead includes heterocycles, terpene-like acids, carotenoids, and long-chain phenolics. The highly abundant Mol1 structure contains multiple hydroxyl groups and a carboxylic acid, which together support strong hydrogen-bond donor and acceptor capacity and may facilitate interactions with polar enzyme pockets. The quinolinone-related Mol8 scaffold combines an aromatic heterocycle with a hydroxylated unsaturated side chain, creating a balance between π-surface interactions and polar anchoring that can stabilize binding in mixed hydrophobic–polar sites. Mol7 incorporates additional oxygenation, including methoxy and hydroxyl functionalities, which can increase binding specificity through directional hydrogen bonding and electrostatic complementarity. The carbazole-dione motif in Mol6 is a rigid, conjugated system that can participate in π–π stacking and may support redox-linked activity due to its quinone-like carbonyl features. In contrast, Mol5 represents a terpene-derived acid framework with bulky hydrophobic volume, suggesting potential affinity for lipid environments and hydrophobic protein grooves, while the carboxyl group can still provide a polar interaction handle. β-Carotene contributes an extended polyene chain lacking heteroatoms, implying that its primary biological contribution is more likely antioxidant quenching and membrane-associated radical buffering rather than specific hydrogen-bond–driven target binding. The long-chain phenolic metabolites Mol12 and Mol13 combine a phenolic ring with C_17_–C_19_-like hydrophobic tails, a pattern that can promote membrane partitioning while retaining phenolic redox activity at the interface. Mol14 includes both a hydroxypiperidine moiety and an extended unsaturated chain, suggesting amphiphilic behavior that may influence cellular uptake and interaction with membrane-proximal targets. The presence of a sphingoid base-like compound (Mol4) also implies potential modulation of lipid-related processes because sphingolipid scaffolds are frequently linked to cell survival and stress responses. The coexistence of polar acids and strongly lipophilic structures indicates that the extract may simultaneously engage aqueous-phase enzymes and membrane-associated signaling components. This duality can be advantageous when biological activity requires both antioxidant buffering and modulation of signaling cascades. The abundance-weighted profile suggests that Mol1, Mol8, Mol7, and Mol5 are priority candidates for downstream mechanistic assays because they combine high representation with chemically interactive functional groups. At the same time, β-carotene and the long-chain phenolics may contribute supportive antioxidant and membrane effects that shape the biological response environment. Taken together, the structural heterogeneity provides a plausible chemical basis for multi-pathway bioactivity derived from the *Averrhoa bilimbi* L. leaf ethanolic extract.

### 3.3 Pharmacokinetic and drug-likeness assessment of *Averrhoa bilimbi* L. leaf metabolites

The *in silico* ADME and drug-likeness evaluation reveals substantial variability in the pharmacokinetic behavior of metabolites identified from *Averrhoa bilimbi* L. leaf ethanolic extract. As summarized in Table 3, most compounds exhibited high predicted gastrointestinal absorption, indicating favorable oral uptake potential across a broad chemical space. High GI absorption was observed for Mol1 through Mol9, Mol11, and Mol14, suggesting that moderate polarity and balanced lipophilicity dominate the extract composition. Topological polar surface area values for these compounds largely fall below 90 Å², a range commonly associated with efficient intestinal permeability. Mol1, Mol2, Mol3, Mol7, and Mol8 showed particularly balanced TPSA and consensus Log Po/w values, supporting their predicted oral bioavailability. In contrast, Mol10, Mol12, and Mol13 displayed low GI absorption, which correlates with their extreme lipophilicity and limited polarity. Mol10 exhibited a consensus Log P_o/w_ of 11.11 and zero TPSA, indicating strong hydrophobic character and poor aqueous solubility. Such properties are typically unfavorable for oral absorption and systemic distribution. Despite this, highly lipophilic compounds may still contribute to biological activity through membrane-associated mechanisms. BBB permeability predictions indicate that several metabolites, including Mol2, Mol5, Mol7, Mol8, Mol9, and Mol11, may penetrate the central nervous system. This property may be advantageous or disadvantageous depending on the intended therapeutic application. Most compounds were predicted not to be P-gp substrates, suggesting reduced risk of efflux-mediated bioavailability loss. Lipinski’s rule of five analysis showed that the majority of metabolites satisfied the criteria with zero or one violation. These results indicate acceptable drug-like characteristics for many constituents. Bioavailability scores clustered around 0.55 for most compounds, supporting moderate systemic exposure. As a whole, the ADME profile highlights a subset of metabolites with favorable oral drug-likeness.

**Table 3:** In silico ADME and Drug-Likeness Profiles of Selected Metabolites from *Averrhoa bilimbi* L. Leaf Ethanolic Extract.

**No**	**Compound ID**	**Molar Refractivity**	**TPSA**	**Consensus Log P**_**o/w**_	**GI Absorption**	**BBB Permeant**	**P-gp substrate**	**Lipinski**	**Bioavailability Score**	**Leadlikeness**	**Synthetic Accessibility**
1	Mol1	56.97	77.76 Å²	0.90	High	No	No	Yes; 0 violation	0.56	No; 1 violation: MW< 250	3.66
2	Mol2	78.85	61.72 Å²	2.48	High	Yes	No	Yes; 0 violation	0.55	Yes	1.74
3	Mol3	94.91	79.90 Å²	2.87	High	No	Yes	Yes; 0 violation	0.55	Yes	3.79
4	Mol4	94.36	86.71 Å²	3.29	High	No	Yes	Yes; 0 violation	0.55	No; 2 violations: Rotors> 7, XLOGP3 > 3.5	4.42
5	Mol5	94.33	37.30 Å²	4.74	High	Yes	No	Yes; 1 violation: MLOGP> 4.15	0.85	No; 1 violation: XLOGP3 > 3.5	4.74
6	Mol6	110.21	70.16 Å²	4.72	High	No	No	Yes; 0 violation	0.55	No; 2 violations: MW> 350, XLOGP3 > 3.5	3.47
7	Mol7	92.98	77.76 Å²	2.71	High	Yes	No	Yes; 0 violation	0.55	No; 1 violation: XLOGP3 > 3.5	3.04
8	Mol8	88.20	53.09 Å²	3.40	High	Yes	No	Yes; 0 violation	0.55	Yes	3.27
9	Mol9	111.93	38.33 Å²	5.33	High	Yes	No	Yes; 0 violation	0.55	No; 3 violations: MW> 350, Rotors> 7, XLOGP3 > 3.5	3.62
10	Mol10	184.43	0.00 Å²	11.11	Low	No	Yes	No; 2 violations: MW> 500, MLOGP> 4.15	0.17	No; 3 violations: MW> 350, Rotors> 7, XLOGP3 > 3.5	6.19
11	Mol11	91.38	40.46 Å²	3.88	High	Yes	Yes	Yes; 0 violation	0.55	No; 1 violation: XLOGP3 > 3.5	5.10
12	Mol12	122.18	57.53 Å²	6.86	Low	No	No	Yes; 1 violation: MLOGP> 4.15	0.55	No; 3 violations: MW> 350, Rotors> 7, XLOGP3 > 3.5	3.23
13	Mol13	123.33	38.69 Å²	7.46	Low	No	No	Yes; 1 violation: MLOGP> 4.15	0.55	No; 3 violations: MW> 350, Rotors> 7, XLOGP3 > 3.5	3.56
14	Mol14	138.18	60.77 Å²	6.16	High	No	Yes	Yes; 0 violation	0.55	No; 3 violations: MW> 350, Rotors> 7, XLOGP3 > 3.5	4.83

A closer inspection of lead-likeness and synthetic accessibility provides further insight into translational potential. Only Mol2, Mol3, and Mol8 satisfied lead-likeness criteria without major violations, indicating suitability for early-stage drug optimization. Several metabolites failed lead-likeness primarily due to excessive lipophilicity, molecular weight, or high rotatable bond counts. Mol9, Mol10, Mol12, Mol13, and Mol14 showed multiple lead-likeness violations, suggesting challenges in further medicinal chemistry refinement. Nevertheless, lead-likeness filters are conservative and may exclude compounds with significant biological relevance. Synthetic accessibility scores ranged from 1.74 to 6.19, reflecting varied structural complexity. Mol2 exhibited the lowest synthetic accessibility score, indicating relative ease of chemical synthesis or modification. In contrast, Mol10 showed the highest synthetic accessibility score, consistent with its large, highly conjugated structure. Moderate synthetic accessibility values for Mol1, Mol3, Mol6, Mol7, and Mol8 suggest feasible optimization pathways. The combination of acceptable synthetic accessibility and favorable ADME parameters enhances the attractiveness of certain metabolites as pharmacological candidates. Compounds with high lipophilicity but manageable molecular size may still be optimized through structural simplification. In comparison, extremely hydrophobic molecules may serve as inspiration for fragment-based or scaffold-hopping strategies. The balance between polarity and lipophilicity remains a key determinant of drug-likeness in this dataset. Importantly, the ADME screening does not aim to eliminate compounds outright but to prioritize candidates rationally. Several metabolites demonstrate a compromise between drug-likeness and chemical diversity. This balance is particularly relevant for natural product-derived drug discovery. The data support selective advancement of compounds with manageable physicochemical profiles. Thus, ADME profiling serves as a critical filter before mechanistic evaluation.

**Fig. 3:**
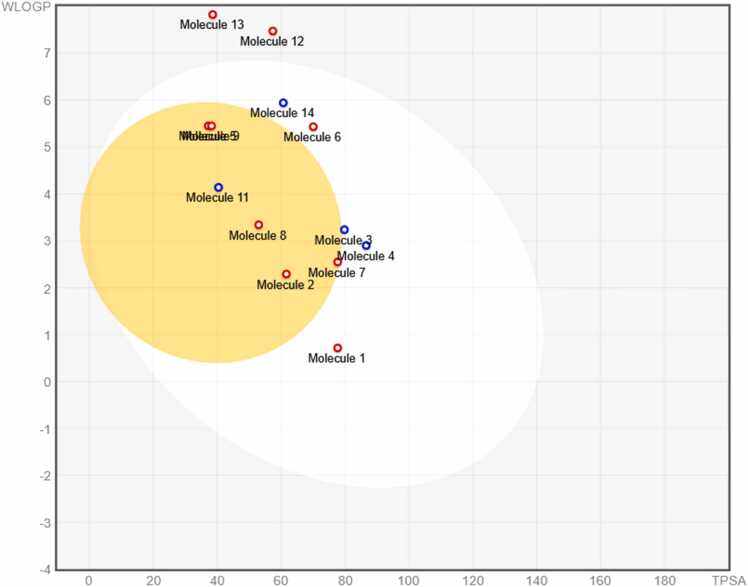
BOILED-Egg Model Plot of Selected Metabolites from *Averrhoa bilimbi* L. Leaf Ethanolic Extract.

The BOILED-Egg model further clarifies absorption and distribution trends among the selected metabolites. As illustrated in Fig. 3, most compounds are positioned within the white region, indicating a high probability of passive gastrointestinal absorption. Several metabolites also fall within the yolk region, suggesting potential BBB permeability consistent with earlier predictions. Compounds located outside both regions reflect poor absorption and limited brain penetration. Mol10, Mol12, and Mol13 are positioned outside the egg, consistent with their low GI absorption and extreme lipophilicity. This visualization confirms that physicochemical extremes strongly influence pharmacokinetic behavior. Compounds within the white region but outside the yolk may offer peripheral activity without central nervous system exposure. Such a profile is often desirable for reducing CNS-related side effects. The BOILED-Egg model also integrates P-gp substrate prediction, which further refines absorption interpretation. Most compounds were classified as non-P-gp substrates, supporting stable intestinal absorption. The concordance between BOILED-Egg results and numerical ADME parameters strengthens confidence in the predictions. The model highlights Mol2, Mol7, and Mol8 as compounds with favorable absorption and distribution balance. These metabolites combine moderate lipophilicity with acceptable polarity. Their positioning suggests suitability for systemic exposure following oral administration. In contrast, compounds outside the egg may require alternative delivery strategies. Such strategies could include formulation enhancement or structural modification. The BOILED-Egg analysis therefore complements conventional ADME metrics. Together, these results delineate clear pharmacokinetic clusters within the metabolite set. This clustering facilitates rational prioritization for downstream biological and mechanistic studies.

### 3.4 Target prediction profiles of *Averrhoa bilimbi* L. leaf metabolites and relevance to antidiabetic mechanisms

Target prediction of the LC–MS/MS-annotated *Averrhoa bilimbi* L. leaf metabolites provides a mechanistic bridge between chemical identity and potential antidiabetic action. Several metabolites were linked to proteins controlling lipid handling, glucose utilization, insulin signaling, and inflammatory tone, which are core axes in Type 2 Diabetes Mellitus progression. The predicted target landscape summarized in Table 4 indicates that Mol1 and Mol2 already span both metabolic regulators and signaling enzymes that are frequently implicated in insulin resistance. Mol1 was associated with lipid-sensing and fatty-acid trafficking machinery such as PPARA, PPARD, and FABP family members, together with FFAR1, suggesting potential modulation of nutrient-responsive transcription and lipid-driven insulin sensitivity. This profile is meaningful because PPAR-centered control of fatty-acid oxidation and lipid storage can influence ectopic lipid accumulation that blunts insulin responsiveness in liver and muscle. Mol1 also mapped to bile acid-related receptors and transport-linked proteins (for example NR1H4, GPBAR1, and NPC1L1), supporting a hypothesis that intestinal lipid handling and bile-acid signaling may be relevant to downstream glycemic control. The presence of AKR1B1 and AKR1B10 in the Mol1 target list is notable because aldo-keto reductases are frequently discussed in the context of glucose-related stress and carbonyl detoxification pathways that intensify under hyperglycemia. Additional Mol1-associated enzymes such as FBP1, G6PD, and ACLY connect to gluconeogenesis, redox homeostasis, and lipogenesis, indicating that predicted activity is not limited to a single metabolic node. From a phytochemical perspective, Mol1 is a small, oxygenated carboxylic acid-like scaffold that may plausibly interface with metabolic enzymes and lipid-binding proteins that accept polar ligands. Mol2, annotated as 4′-O-methylnorbelladine, showed a broader signaling signature that included AKT1 and PIK3CA, alongside multiple kinase regulators (such as JAK1, JAK2, and TYK2), implying possible influence on insulin pathway propagation and cytokine-associated insulin resistance. This matters because AKT1 is a central effector of insulin-stimulated glucose uptake and glycogen synthesis, while PI3K activity is a gateway step that determines whether insulin receptor signaling proceeds effectively. Mol2 also intersected with proteases and modulators such as DPP9 and other regulatory nodes, implying potential crosstalk with peptide metabolism and inflammatory signaling that can reshape glucose homeostasis. The simultaneous appearance of ESR1 and ESR2 among Mol2 targets is also relevant because estrogen receptor signaling is known to affect adiposity distribution and insulin sensitivity, which can modify diabetic risk trajectories. Taken together, Mol1 emphasizes lipid transport and metabolic enzyme control, whereas Mol2 emphasizes kinase signaling and regulatory proteins, creating a mechanistic pairing that aligns well with the multifactorial nature of antidiabetic therapy development.

**Table 4:** Predicted Molecular Targets of *Averrhoa bilimbi* L. Leaf Bioactive Compounds.

**No**	**Prediction Compound Name**	**Compound ID**	**Canonical SMILES**	**Target Prediction**
1	(1 R,2S,3 R,4 R,5 R)-3,4-dihydroxy-5-methyl-2-(propan-2-yl)cyclohexane-1-carboxylic acid	Mol1	O[C@@H]1[C@H]([C@@H](C[C@H]([C@H]1 O)C)C(=O)O)C(C)C	FNTA, FNTB, CHRNA7, NR1H4, GPBAR1, FDFT1, PTGER2, PTGFR, AR, VDR, NPC1L1, POLB, AKR1B10, FABP4, PPARA, FABP3, FABP5, PPARD, FFAR1, FABP2, HSD11B2, UGT2B7, SERPINA6, SHBG, HSD17B3, G6PD, AKR1B1, HSD11B1, GABRA2, GABRB2, GABRG2, HAO1, FBP1, KDM2A, PHF8, CPA1, CDC25A, MME, ACE, ACLY
2	4'-o-methylnorbelladine	Mol2	COC1 =C(C=C(C=C1)CNCCC2 =CC=C(C=C2)O)O	DRD1, DRD3, SIGMAR1, DRD2, KDM1A, MAOA, DRD4, HTR2A, HTR2C, DHCR7, SLC6A3, NQO2, HTR2B, SLC6A4, OPRM1, OPRD1, CASR, SLC6A2, OPRK1, PNMT, MAOB, CA2, CA1, ADRB2, ADRB1, ROCK1, AURKB, RPS6KB1, HTR1A, JAK3, JAK1, JAK2, TYK2, IKBKB, CA12, CA9, TACR1, CHRNB1, CHRNA1, CHRNG, CHRND, CHEK1, ADRA2A, ADRA2C, AADAT, DRD5, NOS1, NQO1, PBK, PRKCA, PRKCQ, PIK3CA, TBXA2R, ADRB3, AKT1, AOC3, ESR1, ESR2, FAP, DPP9, CAMK2D, CSNK1D, GRIK2, CSNK1E, CCNE1, CDK2, PRKCD, PRKCE, PRKCH, ROCK2, PIM1, PIM2, ABAT, CDK2, HRH3, ABCB1, ADRA1D, ADRA1A, PKN1, ACHE, YARS, HASPIN, PRKCB, PRKD1, WEE1, ROCK2, ROCK1, HTR1F, TH, CCR4, PREP, DYRK2, PRMT6, PRKCI, LNPEP, PRMT8, IDO1, PRMT1, CHEK2, ADRA2B, TYRO3
3	Psoralenol	Mol3	CC1(C(CC2 =C(O1)C=CC(=C2)C3 =COC4 =C(C3 =O)C=CC(=C4)O)O)C	ALDH2, CYP19A1, HSP90AA1, ADRB2, ADRB1, HSP90AB1, GCGR, IL2, PTGS1, SLC6A2
4	4-Hydroxy-8-sphingenine	Mol4	CCCCCCCCC/C=C/CCC[C@H]([C@H]([C@H](CO)N)O)O	CYP17A1, S1PR5, S1PR4, S1PR3, S1PR1, ADRB2, SGPL1, ADRB1, S1PR2, LPAR2, DHCR7, GLB1, SPHK1, HRH3, GBA, ALDH2, CYP19A1, HSP90AA1, HSP90AB1, GCGR, IL2, PTGS1, SLC6A2
5	(3S)-5-[(1S,4aR,8aR)-5,5,8a-trimethyl-2-methylidene-3,4,4a,5,6,7,8,8a-octahydronaphthalen-1-yl]-3-methylpentanoic acid	Mol5	C[C@@H](CC[C@H]1 C(=C)C=C[C@H]2[C@@]1(CCCC2(C)C)C)CC(=O)O	NR1H3, PTPN1, PTPN2, PPARG, PPARA, PPARD, FABP4, TOP2A, HSD11B1, PTGES, FFAR1, CES2, PREP, FABP3, SRD5A2, CDC25B, AR, ACP1, AKR1B10, ESR1, ESR2, SCD, FAAH, CDC25A, FABP1, FABP5, ALOX5, CD81, PRKCH, PTPN11, PTPN6, SERPINA6, SHBG, G6PD, CYP51A1, PTGER2, PDE4D, PTGDR2, LTB4R, NPC1L1, SIGMAR1, CYP17A1, PTGS1, TNF, PTGER1, CYP19A1, PTGES2, SLC22A12, SLC6A3, MAPK3, PTGS2, HMGCR, FNTA, FNTB, PTGER4, MDM2, CYP26B1, PGR, BCHE, BACE1, TERT, ALOX12, POLB, NR3C1, HSD11B2, RARG, RARB, TOP1, RORC, RARA, ADORA3, CYP26A1, PSEN2, PSENEN, NCSTN, APH1A, PSEN1, APH1B, PLA2G1B, IMPDH2, RXRA, VDR, CHRM2, ACHE, SLC6A2, CYP2C19, SLC16A1, PTPRF, PTGDR, PTGIR, TP53, RXRG, EDNRA, RORA, CNR1, ENPP2, CCR9, CCR2
6	8-[(2*E*)-3,7-dimethylocta-2,6-dien-1-yl]-7-hydroxy-3-methyl-9H-carbazole-1,4-dione	Mol6	CC(C)=CCC/C(C)=C/Cc1c(O)ccc2c3c([nH]c12)C(=O)C=C(C)C3 =O	HSD17B2, HSD17B1, MTOR, PIK3CA, TBXA2R, PTGES, CTSK, CTSS, CTSL, HSP90AA1, CTSB, HSD11B1, BRD4, BRPF1, MTNR1A, MTNR1B, CXCR2, CXCR1, FASN, HSP90AB1, TYMS, CCR1, MMP13, MMP2, TSPO, ESR1, ESR2, GSK3B, CCKBR, HTR1A, SLC6A4, PDK1, BRD3, AURKB, AURKA, ACACB, PBRM1, HDAC7, MMP3, SMARCA4, MMP9, HDAC1, MMP1, ADAM17, S1PR3, ELANE, MMP14, PDE10A, MMP8, PDF, PTGS1, PTGS2, NPY1R, ADORA1, ADORA2A, ROCK2, EGLN1, MAOA, MAOB, HCRTR2, HCRTR1, CTSV, HDAC3, HDAC6, HDAC2, DRD1, DRD2, HDAC5, HDAC8, MMP7, HDAC10, PTGER2, NR4A1, AR, PDE5A, PYGL, RORC, RAF1, FAAH, MGLL, BRAF, MMP16, DYRK1A, ADAMTS4, MAPK1, MMP12, CCNE2, CDK2, CCNE1, TERT, EP300, ERBB2, EGFR, LCK, PLEC, PRSS1, CTSG, CTRB1, OXTR, CDK2, CCNA1, CCNA2, SMARCA2, MAPK3
7	Methyl 7-hydroxy-3-methoxy-1-methyl-8-(3-methylbut-2-en-1-yl)-4-oxo-1,4-dihydroquinoline-2-carboxylate	Mol7	COC(=O)c1c(OC)c(=O)c2ccc(O)c(CC=C(C)C)c2n1C	KDR, ESR1, ESR2, CCNA2, CDK2, TUBB1, SLC29A1, GRM5, KIF11, RET, MAPK1, EGFR, TACR3, QPCT, CASR, BACE2, MTOR, PIK3CA, BACE1, HDAC6, CCKBR, HDAC1, PRKDC, ATR, ABCB1, PDE4B, HSD17B2, CDK4, HSD11B1, RAF1, QPCT, BRAF, KDM1A, CYP19A1, JAK3, FLT1, PDGFRB, KIT, MET, TSPO, IMPDH2, MAPK10, PIM3, ALOX12, DYRK1B, ROS1, ELANE, PDE2A, ADORA1, ADORA2A, ADORA2B, ADORA3, FGFR1, PARP1, NPY5R, TRPV1, PTGER2, PDE4A, PDE4D, PDE4C, PIM1, MYLK, GRK3, PDE5A, CDK5R1, CDK5, CCNE2, CDK2, CCNE1, CCNB3, CDK1, CCNB1, CCNB2, CDK2, CCNA1, CCNA2, CTSD, CDK2, LDLR, GSTA1, GRK2, GRK5, PIK3R1, GSK3B, LNPEP, EPHB2, CHRNA4, CHRNB2, HSD17B1, ADRA1D, PRKCA, CA7, CA12, CA14, CA9, AURKA, PDK1, ROCK2, ROCK1, SCN2A, ASAH1, CALM1, TBXAS1, DBF4, CDC7, ALOX5, JAK2, HSP90AA1, SLC5A2, CAPN1, LTB4R
8	2-[(4E,6E)-3-hydroxynona-4,6-dien-1-yl]-1H-quinolin-4-one	Mol8	CC/C=C/C=C/C(CCC1 =CC(=O)C2 =CC=CC=C2N1)O	PSEN2, PSENEN, NCSTN, APH1A, PSEN1, APH1B, HSD17B2, HMGCR, TRPA1, PER2, PTGS2, F10, ALOX5, PDE2A, PDE10A, ALOX5AP, OPRL1, CCR1, MMP3, HTR6, CSNK1G2, CSNK1A1, CSNK1D, EPHX2, FAAH, IL6ST, TRPV1, KCNA5, EGFR, ROCK2, ROCK1, GCGR, MAPK14, MAP2K1, SCN9A, KCNK3, MMP9, GRM5, MMP1, ABCB1, JAK3, JAK1, P2RX7, PPARG, MAPK8, TRPV4, BDKRB1, BACE1, NPY5R, LRRK2, MDM4, AGTR1, AKR1C3, MDM2, PIM1, HTR2A, HTR2C, KCNH2, ELANE, PYGL, PIM2, BRAF, PRMT3, SMO, MAOA, HTR1A, GABRA1, TYK2, AVPR2, AVPR1A, PIN1, PDE4D, PLK1, GPR88, EPHX1, KDR, NTRK1, CRHR1, MTNR1A, MTNR1B, CASP3, SYK, CASP6, CASP7, CASP1, MC4R, MTOR, SIGMAR1, PIK3CA, ABCC9, F2, PRKCG, PRKCD, PRKCA, PRKCB, FDFT1, PRKCE, PRKCH, PRKCQ, BRS3, MAP3K20, MAPK11, TGFBR2, TGFBR1, TSPO
9	(4E,7S)-7-methoxy-N-(2-phenylethyl)tetradec-4-enimidic acid	Mol9	CCCCCCC[C@@H](C/C=C/CCC(=O)NCCC1 =CC=CC=C1)OC	CASR, NR1H4, CNR1, HSP90B1, HSP90AA1, HSP90AB1, MDM2, CRHR1, EZH2, MAPK14, BCL2, PIK3CA, SCN9A, TACR2, F10, PDE10A, PRKDC, OPRM1, OPRD1, KCNQ2, TTK, FASN, FNTA, FNTB, CACNA1B, MAPK8, MAPK9, AVPR2, PFKFB3, PIK3CD, PIK3CB, PIK3CG, S1PR3, RORC, NAMPT, UTS2R, GHSR, ERBB2, CCKAR, IGF1R, CSF1R, GCK, HCRTR2, HCRTR1, NR3C1, HTR6, F2R, PTGER1, PDE5A, CDK4, IDH1, PTAFR, PDE4A, NPY5R, MDM4, AGTR1, CFD, MTOR, KIF11, PLAT, SRC, CAMK2D, S1PR2, BCL2L1, GPR119, DGAT1, RAC1, KCNQ1, CXCR2, CDK2, CCNA1, CCNA2, PDE2A, SYK, ACKR3, KDR, CDK2, MAPK1, TEK, CCR8, FLT3, JAK2, TYK2, EPHA2, PIK3CA, PIK3R1, TGFBR1, CD38, ICMT, MAP3K8, RHOA, BAD, SCN2A, P2RY1, SCN10A, PABPC1, INSR, EGFR, JAK3, ADORA2A, JAK1, MAP2K1, SLC33A1, PIK3C2B
10	beta-carotene	Mol10	CC1 =C(C(CCC1)(C)C)/C=C/C(=C/C=C/C(=C/C=C/C=C(/C=C/C=C(/C=C/C2 =C(CCCC2(C)C)C)\C)\C)/C)/C	RBP4
11	(1R,3aR,5aR,7S,9aS,11aR)-1-[(2S)-1-hydroxypropan-2-yl]-3a,6,6,9a,11a-pentamethyl-icosahydro-1H-cyclopenta[*a*]phenanthren-7-ol	Mol11	C[C@@]1(CC[C@@]23 C[C@@H]1 C[C@@H]2CC[C@@H]4[C@@]3(C[C@H](CC4(C)C)O)C)O	AR, ESR1, ESR2, UGT2B7, SHBG, HSD11B1, CDC25B, GPBAR1, SHH, CDC25A, TRPM8, CA2, CA1, NR1I3, CA4, POLA1, NR1H4, CYP19A1, NPC1L1, CYP17A1, PGR, SERPINA6, HSD17B3, NR3C2, NR3C1, SLC6A3, ADORA3, MAPK3, SRD5A1, SRD5A2, G6PD, IDO1, OPRM1, OPRD1, C5AR1, OPRK1, SIGMAR1, HSD11B2, GABBR2, GABBR1, TRPV1, JAK3, JAK1, JAK2, TYK2, HIF1A, EPHX2
12	1-(3,5-dihydroxyphenyl)nonadecan-2-one	Mol12	CCCCCCCCCCCCCCCCCC(=O)CC1 =CC(=CC(=C1)O)O	POLB, IGF1R, SRC, PTK2, ALK, HSD17B1, ESR2, AR, SHBG, NR1H3, NR1H2, KDR, ACHE, HSD17B3, NR3C1, ALOX15, TTR, ALOX12, LTB4R, CNR1, ESR1, SLC6A4, CES2, CYP19A1, ALOX5
13	4-heptadecyl-2,3-dimethoxyphenol	Mol13	CCCCCCCCCCCCCCCCCC1 =C(C(=CC(=C1)O)OC)OC	CETP, HMGCR, CNR1, CNR2, ALOX15, TTR, HTR2A, ALOX12, AKT1, PTGDR2, IGF1R, PTGS2, ALOX5, F3, F7, HTR1A, DRD2, DRD3, ACACB
14	22-hydroxy-1-[(3 R)-3-hydroxypiperidin-1-yl]docosa-2,4-dien-1-one	Mol14	C1C[C@H](CN(C1)C(=O)C=CC=CCCCCCCCCCCCCCCCCCO)O	OPRD1, CNR1, CNR2, RBP4, BCHE, OPRM1, POLB

Beyond the early-numbered metabolites, later compounds expand the antidiabetic mechanistic space by including direct glucose-sensing and insulin-receptor–related targets. Mol9 showed an exceptionally dense target list that includes INSR, IGF1R, and GCK, alongside PI3K pathway members and downstream growth or survival regulators such as MTOR and MAPK1, which are frequently linked to insulin resistance when dysregulated. The inclusion of INSR is particularly important because insulin receptor engagement is the initiating event for canonical glycemic control signaling, and perturbations at this level can translate to systemic hyperglycemia. GCK is a key glucose sensor that influences hepatic glucose handling and pancreatic β-cell responsiveness, so predicted interaction here supports a plausible route for improving glucose-dependent metabolic switching. The presence of MTOR and MAPK-related nodes in the same Mol9 network suggests that this metabolite could influence nutrient sensing and stress-activated signaling that often contributes to impaired insulin action. Mol9 also mapped to receptors and metabolic regulators such as GPR119 and multiple sphingosine-1-phosphate receptors, which is relevant because GPCR-mediated incretin-like signaling and lipid mediator pathways can affect insulin secretion and peripheral sensitivity. In contrast, Mol10 (β-carotene) was mapped specifically to RBP4, a target that is widely discussed as a mediator linking adipose signaling to insulin resistance, making this association mechanistically consistent with antidiabetic framing. At the same time, Mol10 displays physicochemical constraints that can limit oral performance, since it was predicted to have low gastrointestinal absorption and extreme lipophilicity, which can reduce exposure without formulation support. This pattern suggests that Mol10 may be better positioned as a formulation-driven candidate rather than a direct lead compound under conventional oral drug criteria. The divergence between Mol9’s broad signaling coverage and Mol10’s single-target mapping also helps rationalize prioritization, where compounds engaging INSR/GCK/PI3K–mTOR may be advanced for pathway-level validation, while RBP4-directed candidates may be explored for adipose–liver axis modulation. Mechanistically, combining metabolites predicted to influence insulin receptor signaling with others predicted to influence lipid transport and PPAR regulation could better address mixed dyslipidemia and hyperglycemia in Type 2 Diabetes Mellitus. The target maps further imply that *Averrhoa bilimbi* L. metabolites may act through both endocrine-like signaling (INSR, IGF1R) and metabolic enzyme remodeling (FBP1, G6PD, ACLY), which is a useful design logic for botanical-derived antidiabetic candidates.

### 3.5 Identification of overlapping targets and PPI network construction for *Averrhoa bilimbi* L. in type 2 diabetes mellitus

**Fig. 4:**
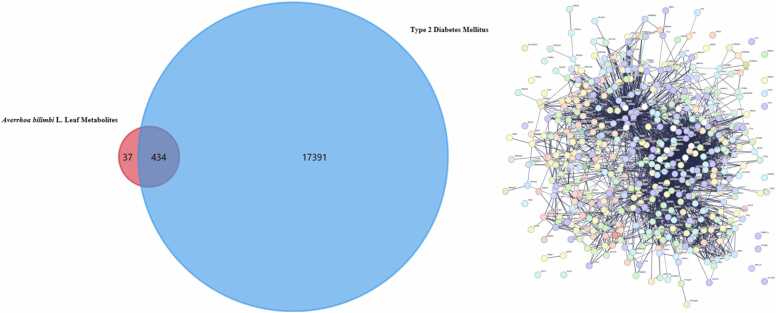
Overlapping Targets and PPI Network Between *Averrhoa bilimbi* L. Leaf Metabolites and Type 2 Diabetes Mellitus.

The integration of *Averrhoa bilimbi* L. leaf metabolites with Type 2 Diabetes Mellitus–related genes revealed a focused intersection that is biologically meaningful despite the extensive pool of disease-associated targets. In this analysis, 434 predicted protein targets were associated with metabolites identified from *Averrhoa bilimbi* L. leaves, while 17,391 genes were linked to Type 2 Diabetes Mellitus. From this large dataset, only 37 overlapping targets were identified, indicating a selective interaction between plant metabolites and diabetes-related molecular mechanisms. This selective overlap suggests that *Averrhoa bilimbi* L. metabolites do not act broadly on unrelated biological processes. Instead, they preferentially target proteins that are directly involved in glucose metabolism, insulin signaling, and metabolic regulation. Such specificity is advantageous for the development of antidiabetic agents, as it reduces the likelihood of off-target effects. The limited number of overlapping targets reflects the complexity of Type 2 Diabetes Mellitus as a multifactorial disease. These shared targets likely represent key regulatory points where metabolic imbalance and insulin resistance converge. The intersection between plant-derived targets and disease genes provides a mechanistic rationale for the traditional use of *Averrhoa bilimbi* L. in metabolic disorders. This relationship is visually summarized in Fig. 4, which highlights the contrast between the extensive disease gene pool and the relatively focused set of intersecting targets. The overlapping genes serve as a refined subset for downstream network-based analysis. Their identification supports the hypothesis that *Averrhoa bilimbi* L. metabolites may exert antidiabetic effects through targeted molecular modulation. This filtering process strengthens the biological relevance of subsequent protein–protein interaction analysis. The intersecting targets represent a critical link between phytochemical diversity and disease-specific signaling pathways. Such integration is essential for translating phytochemical data into therapeutic insights. The overlap analysis therefore provides a strong foundation for network pharmacology–based evaluation of antidiabetic potential.

Further functional insight into these overlapping targets is obtained through protein–protein interaction network construction. The PPI network demonstrates that the intersecting targets are highly interconnected rather than functioning as isolated proteins. Dense connectivity within the network suggests coordinated regulation of metabolic and signaling pathways relevant to Type 2 Diabetes Mellitus. Proteins within this network are likely involved in insulin sensitivity, glucose uptake, inflammatory responses, and lipid metabolism. The presence of highly connected nodes indicates the existence of hub proteins that may play dominant roles in disease progression. Such hubs often act as signal integrators that amplify biological responses when modulated. The interconnected nature of the network supports a multitarget mode of action for *Averrhoa bilimbi* L. metabolites. This characteristic aligns well with the complex pathophysiology of Type 2 Diabetes Mellitus, which involves multiple dysregulated pathways. Simultaneous modulation of several interacting targets may enhance therapeutic efficacy compared to single-target approaches. The PPI architecture also provides a rational basis for prioritizing key proteins for molecular simulation studies. Hub proteins identified from this network are expected to exhibit higher biological impact when targeted. Their modulation may influence multiple downstream processes related to glucose homeostasis. This network-driven prioritization improves the efficiency of *in silico* validation strategies. The PPI analysis complements the overlap analysis by adding functional context to the shared targets. Together, these findings support the potential of *Averrhoa bilimbi* L. leaf metabolites as candidates for antidiabetic drug discovery.

### 3.6 Functional enrichment analysis of *Averrhoa bilimbi* L. leaf metabolite targets related to type 2 diabetes mellitus

**Fig. 5:**
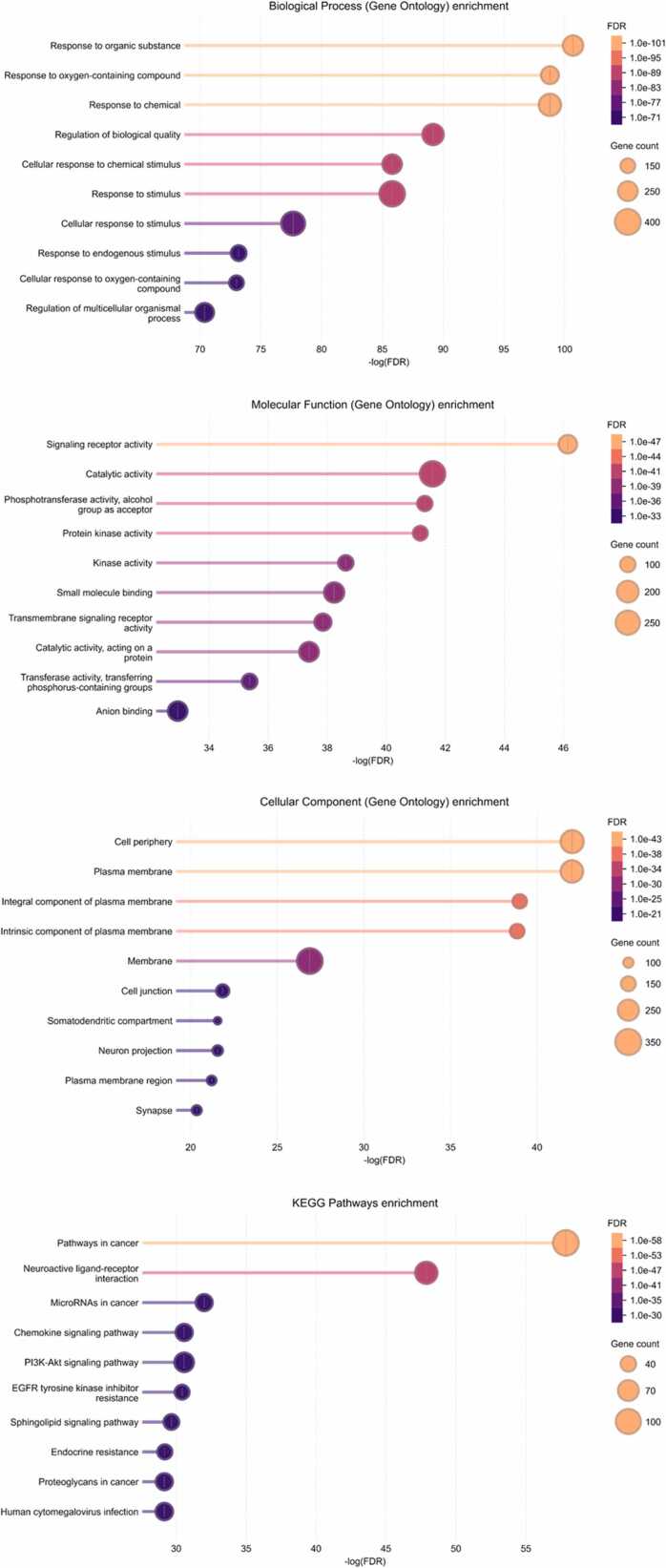
Gene Ontology (BP, MF, and CC) and KEGG Pathway Enrichment Analysis of Overlapping Targets Between *Averrhoa bilimbi* L. Leaf Metabolites and Type 2 Diabetes Mellitus–Related Genes.

The functional enrichment analysis provides mechanistic insight into how *Averrhoa bilimbi* L. leaf metabolites may influence Type 2 Diabetes Mellitus–related molecular processes through their shared targets. As illustrated in Fig. 5, Gene Ontology biological process enrichment highlights strong associations with responses to organic substances, oxygen-containing compounds, and chemical stimuli, which are closely linked to metabolic stress and dysregulated glucose homeostasis in diabetic conditions. The enrichment of cellular response to chemical and endogenous stimuli suggests that the identified targets participate in sensing and adapting to fluctuating metabolic environments. Regulation of biological quality also emerged as a prominent term, reflecting processes involved in maintaining cellular integrity under chronic hyperglycemic stress. Several enriched processes are directly relevant to insulin resistance, including cellular response to stimulus and regulation of multicellular organismal processes. The prominence of oxidative and chemical response pathways aligns with the known role of oxidative stress in the progression of Type 2 Diabetes Mellitus. These findings indicate that the overlapping targets are involved in adaptive and stress-responsive mechanisms rather than isolated metabolic reactions. The enrichment pattern supports the hypothesis that *Averrhoa bilimbi* L. metabolites may modulate upstream regulatory processes affecting glucose metabolism. Cellular signaling-related processes were also significantly enriched, suggesting broad regulatory influence. Such signaling modulation is critical in insulin-sensitive tissues such as liver, muscle, and adipose tissue. The biological process profile reflects a systems-level response to metabolic imbalance. This enrichment landscape reinforces the relevance of the identified targets to diabetes-associated cellular dysfunction. The results suggest that the metabolites act on pathways governing metabolic adaptability. These adaptive processes are central to preventing the progression from insulin resistance to overt diabetes. The biological process enrichment therefore supports the antidiabetic potential of *Averrhoa bilimbi* L. at the molecular level.

Analysis of molecular function and cellular component enrichment further clarifies the biochemical roles of the overlapping targets involved in Type 2 Diabetes Mellitus. Molecular function enrichment reveals dominant roles for signaling receptor activity, catalytic activity, protein kinase activity, and phosphotransferase activity, all of which are fundamental to insulin signaling cascades. The enrichment of kinase-related functions highlights the involvement of phosphorylation-driven signaling, particularly within pathways regulating glucose uptake and insulin sensitivity. Small-molecule binding and transmembrane signaling receptor activity further indicate that these targets act as molecular hubs integrating extracellular metabolic signals. From a cellular component perspective, plasma membrane and intrinsic plasma membrane components were strongly enriched, underscoring the importance of membrane-localized receptors and transporters in glucose regulation. Cell junction and membrane-associated structures were also represented, suggesting roles in intercellular communication relevant to metabolic coordination. KEGG pathway enrichment emphasizes pathways in cancer, PI3K–Akt signaling, EGFR tyrosine kinase inhibitor resistance, and endocrine resistance, which are increasingly recognized as overlapping with metabolic disease mechanisms. The PI3K–Akt signaling pathway is particularly relevant, as it represents a central axis in insulin-mediated glucose transport. Enrichment of sphingolipid and chemokine signaling pathways further supports involvement in inflammatory and lipid-related aspects of diabetes. The appearance of endocrine resistance pathways reflects mechanisms that may underlie impaired insulin responsiveness. These enriched pathways collectively indicate that the overlapping targets occupy critical positions in interconnected metabolic and signaling networks. The functional profile suggests multitarget modulation rather than single-enzyme inhibition. Such polypharmacological characteristics are advantageous for addressing the complex pathophysiology of Type 2 Diabetes Mellitus. The enrichment results therefore provide strong functional support for the proposed antidiabetic activity of *Averrhoa bilimbi* L. leaf metabolites.

### 3.7 Hub gene interaction analysis of the PI3K–AKT signaling pathway in type 2 diabetes mellitus

**Fig. 6:**
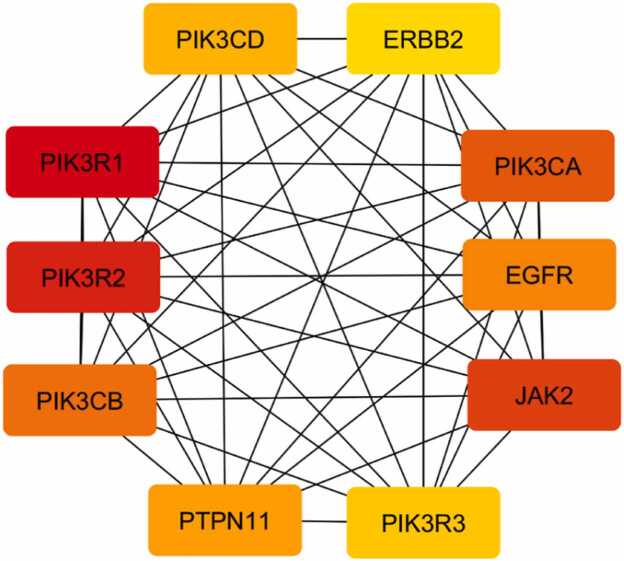
Hub Gene Interaction Network of Key PI3K–AKT Signaling Proteins in Type 2 Diabetes Mellitus.

The hub gene interaction network reveals a highly interconnected structure among key proteins within the PI3K–AKT signaling pathway that plays a central role in the pathogenesis of Type 2 Diabetes Mellitus. The complexity of this network reflects the multifactorial regulation of insulin signaling and glucose homeostasis at the molecular level. Core PI3K subunits, including PIK3R1, PIK3R2, PIK3CA, PIK3CB, and PIK3CD, form a tightly integrated regulatory axis governing intracellular metabolic signal transduction. Strong interconnections among these subunits indicate that PI3K activation is a coordinated process dependent on balanced interactions between catalytic and regulatory components. PIK3R1 and PIK3R2 function as key adaptor proteins that link receptor tyrosine kinases to downstream AKT activation. The involvement of PIK3CA and PIK3CB highlights the dominance of class I PI3K signaling in peripheral glucose metabolism. Notably, the presence of PIK3CD suggests a potential contribution of immunometabolic pathways to insulin resistance. The network also emphasizes EGFR and ERBB2 as critical receptor tyrosine kinases mediating crosstalk between growth and metabolic signaling. Interactions between EGFR and PI3K components suggest mechanistic overlap between proliferative signaling and metabolic dysregulation. JAK2 is incorporated into the network, underscoring the link between cytokine signaling, inflammation, and glucose regulation. PTPN11 appears as a regulatory phosphatase that balances phosphorylation events within the pathway. The high density of connections among nodes indicates that the PI3K–AKT pathway operates as a robust yet tightly regulated signaling system. Such architecture explains why perturbation of a single component may propagate widespread metabolic consequences. This interaction pattern supports the concept that diabetes is driven by coordinated network disturbances rather than isolated molecular defects. The molecular organization of this system is visually illustrated in Fig. 6, highlighting the centrality of PI3K–AKT–associated proteins. The structural integrity of the network suggests both signaling stability and susceptibility to dysregulation. These observations provide a strong molecular framework for interpreting downstream network pharmacology results. The identified hub interactions establish the biological relevance of targeting this pathway in antidiabetic strategies.

From a pharmacological perspective, the dominance of the PI3K–AKT pathway within this network reinforces its suitability as a therapeutic target for diabetes intervention. Simultaneous modulation of multiple nodes may produce more stable metabolic effects than single-target inhibition. The presence of several PI3K subunits indicates that partial regulation across different components could reduce compensatory signaling mechanisms. The integration of EGFR and ERBB2 further suggests that metabolic signaling intersects with growth-related pathways under diabetic conditions. This is particularly relevant given the association between Type 2 Diabetes Mellitus and increased cellular stress and tissue dysfunction. The inclusion of JAK2 highlights the contribution of chronic low-grade inflammation to insulin resistance. Crosstalk between JAK2 and PI3K suggests that immune signaling may directly influence metabolic responsiveness. PTPN11 serves as a critical checkpoint protein that fine-tunes pathway activation through dephosphorylation. The dense connectivity of the hub network supports a systems-level view of diabetes as a complex signaling disorder. Such complexity favors multitarget therapeutic approaches rather than single-molecule interventions. Bioactive metabolites from *Averrhoa bilimbi* L. leaves may exert antidiabetic effects by concurrently modulating several key proteins within this network. This mechanism aligns with the polypharmacological nature commonly observed in plant-derived compounds. Network-level modulation may allow adaptive therapeutic responses to metabolic imbalance. This approach could also reduce adverse effects associated with excessive inhibition of a single signaling node. Hence, the PI3K–AKT pathway emerges as a central molecular axis linking *Averrhoa bilimbi* L. metabolites to glucose regulation. The network analysis strengthens the mechanistic rationale for developing *Averrhoa bilimbi* L. as a natural antidiabetic candidate.

### 3.8 Oil phase selection and surfactant–cosurfactant screening for lipid-based formulation development

The solubility characteristics of *Averrhoa bilimbi* L. leaf extract across different oil phases reveal important formulation-relevant differences that influence subsequent delivery performance. The solubility pattern summarized in Table 5 indicates that oil selection plays a decisive role in determining extract incorporation efficiency. VCO exhibited limited compatibility with the extract, as reflected by consistent precipitate formation at low to moderate extract loadings. This behavior suggests insufficient molecular interaction between VCO and the predominantly polar to semi-polar constituents of the extract. Such incompatibility may hinder uniform dispersion and compromise formulation stability. In contrast, oleic acid demonstrated a greater ability to maintain the extract in a solubilized state at comparable concentrations. This observation implies stronger physicochemical affinity between oleic acid and extract components. Oleic acid possesses amphiphilic properties that facilitate interaction across a wider polarity spectrum. These properties are particularly advantageous for accommodating complex plant extracts. When extract loading exceeded a certain threshold, precipitation occurred even in oleic acid. This phenomenon reflects the existence of a finite solubility capacity that must be respected during formulation design. Concentration-dependent solubility therefore emerges as a critical parameter. Excessive extract loading may destabilize the oil phase and impair downstream emulsification. The observed solubility trends highlight the importance of balancing oil selection with extract concentration. Oleic acid offers a practical solubilization window suitable for formulation development. Such balance is essential for maintaining thermodynamic and kinetic stability. Oil phase optimization thus represents a foundational step in lipid-based system design. These findings establish a rational basis for advancing oleic acid to subsequent emulsification studies.

**Table 5:** Solubility Observation of *Averrhoa bilimbi* L. Leaf Extract in Different Oil Phases.

**Extract Amount**	**Oil Type**	**Observation**
50 mg	VCO	Presence of extract precipitate
50 mg	Oleic acid	No extract precipitate observed
100 mg	VCO	Presence of extract precipitate
100 mg	Oleic acid	No extract precipitate observed
150 mg	VCO	Presence of extract precipitate
150 mg	Oleic acid	Presence of extract precipitate

Beyond oil selection, emulsification behavior further defines formulation quality through stability and dispersion characteristics. The surfactant and cosurfactant screening results presented in Table 6 reveal that all tested systems exhibit acceptable resistance to phase separation. This indicates that the selected emulsifiers are broadly compatible with oleic acid and the extract. However, physical stability alone does not fully capture formulation performance. Differences in optical clarity provide additional insight into emulsification efficiency. Systems formulated with Tween 20 consistently showed lower clarity, suggesting less effective interfacial coverage. Tween 80-based systems, by contrast, produced more uniform dispersions. This difference reflects the higher hydrophilic–lipophilic balance of Tween 80, which favors oil-in-water emulsification. The choice of cosurfactant further modulated system behavior. Propylene glycol improved dispersion clarity relative to PEG 400. This effect may be attributed to enhanced interfacial flexibility and reduced interfacial tension. The Tween 80–propylene glycol combination yielded the most homogeneous dispersion. High clarity is commonly associated with finer droplet size and improved dispersion uniformity. Such characteristics are critical for consistent drug delivery performance. These observations underscore that emulsifier selection directly influences formulation microstructure. Stability and clarity must therefore be evaluated together. A formulation that is stable but poorly dispersed may still underperform. The screening results highlight the importance of selecting emulsifiers that optimize both parameters.

**Table 6:** Surfactant and Cosurfactant Screening Based on Centrifugation Stability and Clarity.

**Oil Type**	**Surfactant**	**Cosurfactant**	**Centrifugation Test Result**	**Clarity**
Oleic acid	Tween 20	PEG 400	No phase separation	+
Oleic acid	Tween 20	Propylene glycol	No phase separation	+ +
Oleic acid	Tween 80	PEG 400	No phase separation	+
Oleic acid	Tween 80	Propylene glycol	No phase separation	+ ++

Notes:

+ (Low clarity): Slightly clear, translucent, and homogeneous.

+ + (Moderate clarity): Fairly clear, translucent, and homogeneous.

+ ++ (High clarity): Clear, transparent, and homogeneous.

From a formulation perspective, the superior performance of the Tween 80–propylene glycol system reflects favorable interfacial dynamics during emulsification. Tween 80 supports spontaneous formation of oil-in-water dispersions due to its high hydrophilic character. This property facilitates rapid self-emulsification upon dilution. Propylene glycol complements this behavior by acting as a co-solvent that enhances interfacial mobility. Together, these components promote efficient droplet breakup and stabilization. Smaller droplets typically translate into higher optical clarity. Enhanced dispersion uniformity may improve dissolution behavior of incorporated bioactives. In contrast, PEG 400 appears less effective in supporting similar interfacial restructuring. Differences in molecular flexibility and solvent polarity may underlie this limitation. Although all systems resisted macroscopic phase separation, clarity differences reveal subtle but important distinctions in dispersion quality. High-quality dispersions are particularly relevant for oral lipid-based delivery systems. Improved dispersion may increase the effective surface area for release of phytochemicals. This effect can enhance absorption potential following administration. For plant-derived extracts, such improvement is especially valuable given their complex composition. The selected surfactant–cosurfactant system therefore provides a robust platform for further optimization. Subsequent steps may include S_mix_ ratio refinement. Construction of a pseudoternary phase diagram would allow systematic exploration of formulation space. These steps aim to maximize formulation robustness and performance. The present formulation screening thus supports the development of *Averrhoa bilimbi* L. as a promising candidate for lipid-based antidiabetic delivery.

### 3.9 Optimization of SNEDDS formulation parameters for *Averrhoa bilimbi* L. leaf extract

The optimization results of the SNEDDS system, as summarized in Table 7, demonstrate that variations in the oil-to-S_mix_ ratio and surfactant–cosurfactant proportion markedly influenced emulsification performance and optical clarity. Formulations with an oil:S_mix_ ratio of 1:7 generally exhibited lower transmittance values and longer emulsification times, indicating suboptimal self-emulsification efficiency. Increasing the S_mix_ proportion to an oil:S_mix_ ratio of 1:8 produced moderate improvements, although several formulations still fell into grade C due to slower emulsification kinetics. A more pronounced enhancement was observed at an oil:S_mix_ ratio of 1:9, where transmittance values exceeded 78% in multiple formulations. High transmittance reflects the formation of finer nanoemulsion droplets, which is a critical indicator of SNEDDS performance. Emulsification times also decreased substantially at higher S_mix_ ratios, suggesting more rapid dispersion upon aqueous dilution. Formulations F8 and F9 exhibited the shortest emulsification times, both below 50 s, which is desirable for oral delivery systems. The grade A classification assigned to these formulations indicates superior clarity and stability compared with other tested ratios. Differences between S_mix_ ratios of 2:1 and 3:2 further reveal the importance of balancing surfactant and cosurfactant concentrations. Excessive surfactant content did not always translate to better performance, highlighting the need for compositional optimization. The presence of Tween 80 and propylene glycol at appropriate proportions facilitated efficient interfacial tension reduction. These findings indicate that emulsification efficiency depends on synergistic interactions rather than individual component dominance. The observed trends align with the fundamental principles of SNEDDS design. Such optimization is essential to ensure reproducible nanoemulsion formation. Improved dispersion characteristics are expected to enhance drug dissolution. This step is therefore critical before extract incorporation. The data provide a rational basis for selecting the optimal formulation window.

**Table 7:** Optimization of Oil (Oleic Acid) and S_mix_ (Tween 80:Propylene Glycol) Ratio in SNEDDS Formulation.

**Formula**	**Oil:S**_**mix**_**Ratio**	**S**_**mix**_**Ratio (Surfactant:Cosurfactant)**	**Transmittance (%)**	**Emulsification Time (s)**	**Grade**
F1	1:7	1:1	55.5	111.8	C
F2	1:7	2:1	70.4	124.7	B
F3	1:7	3:2	71.0	90.6	B
F4	1:8	1:1	67.7	50.41	C
F5	1:8	2:1	66.3	118	C
F6	1:8	3:2	73.8	70.4	B
F7	1:9	1:1	70.3	48.1	B
F8	1:9	2:1	83.2	47.1	A
F9	1:9	3:2	78.6	45.1	A

Building on the optimization stage, Table 8 presents the final SNEDDS composition incorporating the ethanolic extract of *Averrhoa bilimbi* L. leaves. The selected formulation (F8a) was derived from the best-performing oil:S_mix_ ratio identified previously. Oleic acid was chosen as the oil phase due to its favorable solubilization capacity and compatibility with lipid-based systems. Tween 80 served as the primary surfactant, providing strong emulsifying properties and promoting nano-sized droplet formation. Propylene glycol functioned as the cosurfactant, enhancing interfacial flexibility and accelerating self-emulsification. The inclusion of 100 mg of extract demonstrates the system’s capacity to accommodate a relatively high phytochemical load. Maintaining clarity after extract incorporation indicates that the SNEDDS system remained thermodynamically stable. This suggests that the bioactive constituents were efficiently solubilized within the lipid matrix. The selected component ratios preserved rapid emulsification behavior despite the presence of solid extract material. Such performance is essential for ensuring consistent bioavailability upon oral administration. The formulation design minimizes the risk of precipitation during dilution in gastrointestinal fluids. Stability at this stage supports the suitability of the system for further evaluation. The compositional simplicity also favors scalability and reproducibility. Importantly, the use of pharmaceutically accepted excipients enhances translational potential. The formulation strategy reflects a balance between performance and practicality. Incorporation of the extract did not compromise nanoemulsion integrity. This indicates good compatibility between the plant extract and lipid components. The optimized composition provides a robust delivery platform.

**Table 8:** SNEDDS Formulation Containing Ethanolic Extract of *Averrhoa bilimbi* L. Leaves (F8a).

**Component**	**Amount**
Extract (mg)	100
Oleic acid (mL)	1
Tween 80 (mL)	6
Propylene glycol (mL)	3

From a biopharmaceutical perspective, the optimized SNEDDS formulation offers significant advantages for improving the oral delivery of *Averrhoa bilimbi* L. leaf extract. Rapid emulsification and high transmittance are closely associated with increased surface area for dissolution. This property is particularly relevant for phytochemicals with limited aqueous solubility. Enhanced dispersion is expected to promote faster and more consistent absorption in the gastrointestinal tract. The nano-sized droplets formed by the optimized system may facilitate lymphatic transport. Such a pathway can reduce first-pass metabolism and improve systemic exposure. The use of oleic acid may further support absorption by modulating intestinal permeability. Tween 80 is known to enhance solubilization and may also influence efflux transporter activity. Propylene glycol contributes to formulation flexibility and dispersion stability. Together, these components create a synergistic delivery system. The optimized formulation aligns with the objective of maximizing bioavailability without chemical modification of the extract. This is especially important for preserving the integrity of complex phytochemical mixtures. Improved delivery efficiency supports the exploration of antidiabetic potential observed in earlier network pharmacology analyses. Enhanced bioavailability may translate to stronger *in vivo* efficacy at lower doses. The SNEDDS system thus bridges formulation science with pharmacological relevance. It provides a technological solution to overcome solubility-related limitations. The optimized formulation represents a critical step toward functional oral dosage development. These results justify subsequent characterization and biological evaluation.

### 3.10 In vitro performance and physicochemical characteristics of the optimized SNEDDS formulation

The *in vitro* characterization outcomes of the optimized SNEDDS formulation are presented in Table 9, providing a detailed assessment of its physicochemical performance following extract incorporation. High optical clarity was demonstrated by a transmittance value of 81.5%, indicating efficient nanoemulsion formation upon aqueous dilution. Such clarity reflects minimal light scattering, a feature commonly associated with nanoscale droplet systems. Droplet size analysis revealed an average particle diameter of 157.7 ± 4.9 nm, confirming the successful formation of a nano-sized dispersion. Droplets within this size range are favorable for increasing interfacial surface area and enhancing dissolution behavior. The relatively narrow size distribution further supports formulation homogeneity. This observation is reinforced by the polydispersity index value of 0.405 ± 0.067, which falls within an acceptable range for SNEDDS formulations. A moderate PDI suggests controlled droplet formation without excessive aggregation. Agreement between high transmittance and nanoscale droplet size reflects efficient self-emulsification dynamics. These characteristics indicate that the lipid–surfactant system effectively stabilized the extract-loaded droplets. The nanoemulsion properties suggest rapid dispersion in gastrointestinal fluids. Efficient dispersion is essential for maintaining solubilization of phytochemicals after oral administration. The absence of turbidity further supports uniform droplet distribution. Together, these physicochemical parameters indicate that the formulation process achieved the intended performance objectives. Importantly, extract loading did not compromise droplet formation. The system retained its nanoemulsion integrity after dilution. This finding demonstrates good compatibility between the *Averrhoa bilimbi* L. extract and the SNEDDS components. The observed droplet characteristics reflect a rationally designed delivery system. These results establish a strong foundation for subsequent performance evaluation.

**Table 9:** *In vitro* Characterization Results of SNEDDS Containing Ethanolic Extract of *Averrhoa bilimbi* L. Leaves.

**Type of Characterization**	**Parameter**	**Result**
Clarity	Transmittance (%)	81.5%
Droplet size analysis	Particle size (nm)	157.7 ± 4.882 nm
Droplet size distribution analysis	Polydispersity index (PDI)	0.405 ± 0.067
Dispersibility test	Emulsification time (s)	57.48
Dispersibility test	Grade	A
Robustness test	Distilled water	No phase separation
Robustness test	0.1 N HCl	No phase separation
Robustness test	Phosphate buffer pH 6.8	No phase separation
Thermodynamic stability test	Centrifugation	Clear, no phase separation
Thermodynamic stability test	Heating–cooling cycle	Clear, no phase separation
Thermodynamic stability test	Freeze–thaw	Clear, no phase separation

Beyond droplet characteristics, dispersibility and stability testing provided critical insight into formulation robustness. The emulsification time of 57 s indicates rapid self-emulsification under gentle agitation conditions. Rapid emulsification is a key requirement for oral SNEDDS to ensure prompt dispersion after ingestion. The formulation achieved grade A dispersibility, reflecting clear and homogeneous emulsion formation. This grading confirms superior emulsification behavior compared with lower-grade systems. Robustness testing further demonstrated formulation resilience under various aqueous environments. No phase separation was observed in distilled water, indicating strong interfacial stabilization. Stability was also maintained in 0.1 N HCl, simulating acidic gastric conditions. The absence of phase separation in phosphate buffer pH 6.8 suggests reliable performance in intestinal environments. These findings highlight the adaptability of the formulation across physiologically relevant media. Thermodynamic stability testing reinforced these observations. Centrifugation did not induce creaming or separation, indicating resistance to gravitational stress. Heating–cooling cycles showed that temperature fluctuations did not destabilize the system. Freeze–thaw testing further confirmed structural integrity under extreme conditions. Such stability suggests that the nanoemulsion system is thermodynamically favorable rather than kinetically trapped. Strong interfacial films are likely responsible for preventing droplet coalescence and phase inversion. This robustness is critical for storage, transport, and long-term usability. The *in vitro* findings demonstrate that the optimized SNEDDS formulation exhibits rapid performance alongside high physicochemical stability. These attributes support its suitability as an oral delivery system for *Averrhoa bilimbi* L. leaf extract.

### 3.11 In vitro performance and physicochemical characteristics of the optimized SNEDDS formulation

The inhibitory activity of the ethanolic leaf extract and SNEDDS formulations against α-amylase provides important insight into their apparent antidiabetic potential, as summarized in Table 10. The crude ethanolic extract of *Averrhoa bilimbi* L. leaves exhibited a high IC₅₀ value of 440.92 ± 10.96 µg/mL, indicating very weak inhibitory activity toward α-amylase. This result suggests that, in its unformulated state, the extract has limited accessibility or interaction efficiency with the enzyme active site. The large IC₅₀ value may be attributed to poor aqueous solubility of bioactive constituents, leading to reduced effective concentration during the assay. Phytochemicals with low solubility often show diminished enzymatic inhibition despite inherent bioactivity. In contrast, the blank SNEDDS formulation (F8) demonstrated a markedly lower IC₅₀ value of 49.01 ± 1.67 µg/mL. This finding indicates that the lipid-based carrier system itself contributes to enzyme inhibition or enhances interfacial interactions with α-amylase. The strong inhibitory activity of the blank formulation indicates that the carrier cannot be considered pharmacologically inert under the assay conditions. The surfactant-rich environment may facilitate nonspecific enzyme interaction or alter enzyme conformation. However, the most pronounced inhibitory effect was observed for the extract-loaded SNEDDS formulation (F8a). This formulation exhibited an exceptionally low IC₅₀ value of 3.75 ± 0.09 µg/mL, reflecting very strong α-amylase inhibition. Because the blank SNEDDS already exhibited substantial inhibitory activity, the marked reduction in IC₅₀ observed for F8a should not be attributed solely to the encapsulated plant extract. The dramatic improvement in activity compared with the crude extract highlights the role of formulation strategy. Encapsulation within the SNEDDS system likely enhanced solubilization and dispersion of active phytochemicals. Improved dispersion increases the effective surface area available for enzyme interaction. This effect enables bioactive compounds to interact more efficiently with catalytic residues. The narrow standard deviation further indicates consistent inhibitory performance across replicates. Such reproducibility reflects formulation stability during enzymatic testing. At the same time, the current data suggest that the observed inhibition likely arises from the combined contributions of the extract and formulation excipients. These results demonstrate that formulation-dependent effects are critical for antidiabetic evaluation.

**Table 10:** The α-Amylase Inhibitory Activity of Ethanolic Leaf Extract of *Averrhoa bilimbi* L. and SNEDDS Formulations.

**Sample**	**IC₅₀ (µg/mL) – Replicate 1**	**IC₅₀ (µg/mL) – Replicate 2**	**IC₅₀ (µg/mL) – Replicate 3**	**Mean IC₅₀ (µg/mL)**	**Category**
Ethanolic leaf extract of *Averrhoa bilimbi* L.	449.91	428.71	444.16	440.92 ± 10.96	Very weak
Blank SNEDDS (F8)	48.10	47.99	50.94	49.01 ± 1.67	Very strong
Extract-loaded SNEDDS (F8a)	3.74	3.67	3.84	3.75 ± 0.09	Very strong
Acarbose	27.53	24.79	25.82	26.05 ± 1.38	Very strong

A comparative assessment against acarbose, a standard α-amylase inhibitor, further strengthens the interpretation of these findings. Acarbose exhibited a mean IC₅₀ value of 26.05 ± 1.38 µg/mL, which falls within the very strong inhibition category. Although the extract-loaded SNEDDS formulation showed lower IC₅₀ values than acarbose under identical assay conditions, this result should be interpreted cautiously and does not by itself demonstrate superior antidiabetic efficacy. Given the significant activity of the blank SNEDDS, part of the measured inhibition may reflect excipient-driven effects or formulation-related assay interference rather than the intrinsic potency of the plant extract alone. The lipid-based nanoenvironment may facilitate sustained contact between inhibitors and α-amylase. Nano-sized droplets can act as carriers that maintain high local concentrations of active compounds near the enzyme surface. The presence of oleic acid, Tween 80, and propylene glycol may also contribute synergistically to enzyme modulation. These excipients may influence enzyme behavior, substrate accessibility, turbidity, or interfacial properties, all of which could affect absorbance-based measurements. The blank SNEDDS activity indicates that carrier components alone exert a measurable effect, although less potent than the extract-loaded system. Incorporation of the extract appears to amplify this effect through combined physicochemical and biochemical mechanisms. Such synergy aligns with the concept of formulation-driven enhancement, but the relative contribution of phytochemicals and excipients cannot be fully distinguished in the present assay design. The substantial reduction in IC₅₀ following SNEDDS incorporation supports the relevance of delivery systems in antidiabetic development. Improved enzyme inhibition is expected to reduce postprandial glucose release from dietary carbohydrates. This mechanism directly supports the therapeutic rationale for α-amylase inhibition in Type 2 Diabetes Mellitus. Nevertheless, the present findings should be regarded as preliminary evidence of enhanced inhibition within this specific formulation-based assay system rather than conclusive proof of superior pharmacological activity. Further studies involving individual excipient controls, orthogonal enzyme assays, and complementary cellular or *in vivo* validation are required to clarify the true antidiabetic contribution of the extract-loaded SNEDDS. Accordingly, the formulation may be described as a promising system for enhancing apparent α-amylase inhibition, while broader claims regarding antidiabetic superiority should be avoided at this stage. The results also provide limited experimental support for carbohydrate metabolism–related predictions from the network pharmacology analysis, although direct mechanistic validation remains necessary.

## 4 Discussions

The LC–MS/MS profiling of *Averrhoa bilimbi* L. leaf extract revealed a chemically diverse metabolite composition dominated by alkaloids, phenolic derivatives, and other semi-polar constituents, which provides a biochemical basis for its antidiabetic potential. The presence of multiple low- and medium-molecular-weight metabolites supports broad target engagement rather than single-enzyme inhibition. Such chemical diversity is consistent with previous reports describing *Averrhoa bilimbi* L. as a rich source of flavonoids, phenols, and related bioactives with metabolic relevance. However, it should be noted that the identified metabolites were assigned based on LC–MS/MS data without the use of authentic reference standards, and therefore represent putatively annotated compounds rather than fully confirmed chemical identities. Metabolites identified in the extract are known to interact with signaling proteins involved in glucose uptake, insulin sensitivity, and oxidative stress regulation [57]. The network pharmacology analysis further clarified this complexity by identifying a substantial overlap between predicted compound targets and Type 2 Diabetes Mellitus–related genes. These target assignments were derived from *in silico* prediction tools and database integration, and thus should be interpreted as hypothetical associations rather than experimentally validated biological targets. The resulting PPI network demonstrated high connectivity, reflecting the multifactorial nature of diabetic pathophysiology. Key hub proteins were enriched within insulin signaling and inflammatory pathways, indicating coordinated regulation rather than isolated molecular effects. Functional enrichment analysis highlighted biological processes related to responses to organic substances, oxygen-containing compounds, and chemical stimuli, which are closely associated with metabolic stress [58]. KEGG pathway analysis emphasized PI3K–AKT signaling, EGFR-related pathways, and insulin resistance–associated networks. Although these pathways are well-established in glucose homeostasis, their involvement in the present study is inferred from computational analysis and not directly validated through experimental assays. Similar pathway dominance has been reported in *in vivo* studies of *Averrhoa bilimbi* L., where modulation of insulin signaling and glucose transport was observed. The convergence of LC–MS/MS and network pharmacology data supports a multitarget mechanism of action. This systems-level behavior is characteristic of plant-derived antidiabetic agents [59]. Such a mechanism is advantageous in complex metabolic disorders. The integration of chemical and network data strengthens the mechanistic rationale for further formulation development. It also provides molecular justification for downstream biological testing. Together, these findings establish a conceptual link between phytochemical composition and antidiabetic signaling modulation, although this link remains predictive and requires experimental validation.

Previous studies employing molecular docking analysis translated network-level predictions into residue-level interaction evidence by demonstrating favorable binding of selected metabolites to prioritized hub proteins. Several alkaloid-type compounds showed stable interactions with kinases and regulatory proteins involved in insulin signaling. Binding energies within the micromolar to nanomolar range indicate biologically meaningful affinity rather than nonspecific association. These interactions were stabilized through hydrogen bonding and hydrophobic contacts within conserved active or allosteric regions [60]. Proteins associated with the PI3K–AKT signaling pathway, previously identified as key hubs in network pharmacology analysis, emerged as recurrent docking targets, reinforcing their central role in glucose regulation. This convergence between system-level predictions and molecular-level simulations highlights the robustness of the identified targets within insulin signaling networks. Nevertheless, molecular docking provides only theoretical binding predictions and does not confirm functional modulation of these proteins in biological systems. Such integrative patterns are consistent with experimental evidence showing that *Averrhoa bilimbi* L. extracts can enhance insulin sensitivity and glucose utilization in diabetic models. The ability of individual metabolites to interact with multiple targets further supports a polypharmacological mode of action predicted by network pharmacology analysis. This characteristic is particularly relevant for diabetes, where pathway redundancy and compensatory mechanisms often limit the efficacy of single-target therapies. Translating these molecular insights into a delivery strategy, the SNEDDS formulation was designed to address solubility and bioavailability limitations inherent to phytochemicals. Optimization of oil, surfactant, and cosurfactant ratios resulted in rapid emulsification and high optical clarity. Nano-sized droplet formation is expected to increase interfacial surface area and dissolution rate. Improved dispersion facilitates more consistent gastrointestinal absorption. Lipid-based delivery systems are known to enhance lymphatic uptake, which may reduce first-pass metabolism. The selected formulation maintained stability after extract incorporation, indicating good compatibility between the phytochemicals and excipients. However, the direct relationship between prediction-based target associations and *in vivo* bioavailability or pharmacodynamic effects cannot be established within the scope of this study. Such stability is critical for maintaining the integrity of predicted compound–target associations identified through network pharmacology analysis. Formulation science therefore serves as a bridge between *in silico* prediction and biological efficacy [36]. This step ensures that bioactive compounds reach systemic circulation in an effective form. The integration of network pharmacology and formulation data provides a coherent translational framework, although experimental validation at the cellular or organismal level remains necessary.

The antidiabetic relevance of the optimized system was preliminarily assessed through *in vitro* α-amylase inhibition testing, which demonstrated a marked enhancement of activity following SNEDDS incorporation. The crude ethanolic extract exhibited weak inhibitory activity, reflecting limited aqueous solubility and enzyme accessibility. In contrast, the extract-loaded SNEDDS showed a dramatic reduction in IC₅₀ values to the low microgram per milliliter range within the assay system used. However, this improvement should not be interpreted solely as an extract-driven effect, because the blank SNEDDS also exhibited substantial α-amylase inhibition. This observation indicates that the formulation components may contribute to the measured activity. Excipients such as oleic acid, Tween 80, and propylene glycol may influence enzyme inhibition through direct interaction with the enzyme, altered substrate accessibility, or changes in the interfacial environment. Enhanced enzyme inhibition may therefore reflect not only improved delivery of bioactive compounds but also the intrinsic effects of the carrier system. Reduced starch hydrolysis can attenuate postprandial glucose excursions, which is a key therapeutic strategy in Type 2 Diabetes Mellitus. These findings are consistent with earlier *in vivo* studies reporting glucose-lowering effects of *Averrhoa bilimbi* L. extracts via modulation of carbohydrate metabolism [61]. Nevertheless, the present assay design does not allow clear differentiation between the inhibitory contributions of the extract and individual excipients. Improved dispersion likely increased effective concentration at the enzyme interface. In addition, surfactant-based nanoemulsion systems may introduce turbidity, interfacial effects, or colorimetric interference that can influence absorbance-based measurements and potentially lead to overestimation of enzyme inhibition. The stability of the SNEDDS under gastrointestinal-simulating conditions further supports sustained formulation performance after oral administration, but does not directly confirm pharmacological activity in biological systems. Enzyme inhibition may complement the network-predicted modulation of insulin signaling pathways, but direct mechanistic linkage remains to be experimentally validated. The alignment between computational predictions, formulation performance, and biological activity provides supportive but still indirect evidence of antidiabetic potential. Accordingly, the current findings should be interpreted as preliminary evidence of formulation-enhanced α-amylase inhibition rather than definitive proof of pharmacological efficacy. Furthermore, the absence of cellular assays, insulin-signaling validation, glucose uptake studies, or *in vivo* models represents a key limitation of this study. Future studies should include individual excipient controls, solvent and surfactant controls, orthogonal non-colorimetric enzyme assays, as well as cellular and *in vivo* validation to confirm the mechanism of action and therapeutic relevance. Such an approach enables more rigorous and reliable development of plant-based antidiabetic candidates.

## 5 Conclusions

This study demonstrates that *Averrhoa bilimbi* L. leaf extract shows potential antidiabetic relevance based on integrated chemical, computational, formulation, and preliminary biological evaluation. LC–MS/MS analysis revealed the presence of chemically diverse metabolites potentially associated with metabolic regulation, while network pharmacology identified 434 overlapping targets related to Type 2 Diabetes Mellitus, highlighting the possible involvement of the PI3K–AKT and related insulin signaling pathways. These target associations, however, were derived from prediction-based and database-driven analyses and therefore should be interpreted as candidate mechanisms rather than experimentally confirmed molecular effects. In this context, the proposed mechanisms remain hypothesis-generating and require further experimental validation to confirm their biological relevance. Formulation of the extract into a SNEDDS system markedly improved physicochemical performance, producing a nanoemulsion with high transmittance (81.5%), nanoscale droplet size (157.7 ± 4.9 nm), rapid emulsification (57 s), and good stability under the tested conditions. Incorporation of the extract into the SNEDDS system was associated with a substantial reduction in the measured α-amylase IC₅₀ value, from 440.92 ± 10.96 µg/mL for the crude extract to 3.75 ± 0.09 µg/mL for the extract-loaded SNEDDS. However, because the blank SNEDDS also exhibited considerable inhibitory activity, the enhanced inhibition observed for the extract-loaded formulation cannot be attributed solely to the plant extract. The present results therefore indicate stronger inhibition within this assay system, but do not establish superior antidiabetic efficacy or confirm that the observed effect is driven predominantly by the phytochemical constituents. Possible contributions from excipients, interfacial effects, and absorbance-based assay interference should also be considered when interpreting the α-amylase data. Taken together, the findings suggest that SNEDDS formulation can enhance the apparent inhibitory response of *Averrhoa bilimbi* L. extract in an *in vitro* enzyme assay and provide supportive evidence for its further investigation as a natural antidiabetic candidate. Nevertheless, additional studies incorporating excipient-specific controls, orthogonal bioassays, cellular models, and *in vivo* validation are required before definitive conclusions regarding pharmacological efficacy can be made.

## Abbreviations

ADME: Absorption, Distribution, Metabolism, and Excretion
AKT: Protein Kinase B
AMPK: AMP-Activated Protein Kinase
BBB: Blood–Brain Barrier
BP: Biological Process
CC: Cellular Component
CID: Collision-Induced Dissociation
DPP-4: Dipeptidyl Peptidase-4
ESI: Electrospray Ionization
FDR: False Discovery Rate
GI: Gastrointestinal
GLUT: Glucose Transporter
GO: Gene Ontology
KEGG: Kyoto Encyclopedia of Genes and Genomes
LC–MS/MS: Liquid Chromatography–Tandem Mass Spectrometry
MCC: Maximal Clique Centrality
MF: Molecular Function
MS: Mass Spectrometry
MS/MS: Tandem Mass Spectrometry
MSI: Metabolomics Standards Initiative
OMIM: Online Mendelian Inheritance in Man
PDB: Protein Data Bank
PI3K: Phosphoinositide 3-Kinase
PPI: Protein–Protein Interaction
SNEDDS: Self-Nanoemulsifying Drug Delivery System
S_mix_: Surfactant and Cosurfactant Mixture
STRING: Search Tool for the Retrieval of Interacting Genes/Proteins
TPSA: Topological Polar Surface Area
UHPLC: Ultra-High-Performance Liquid Chromatography

## CRediT authorship contribution statement

**Ratih Aryani:** Writing – review & editing, Writing – original draft, Visualization, Validation, Supervision, Software, Resources, Project administration, Methodology, Investigation, Funding acquisition, Formal analysis, Data curation, Conceptualization. **Sarah Sarah:** Visualization, Software, Resources, Methodology, Data curation. **Umi Yuniarni:** Writing – original draft, Visualization, Validation, Supervision, Software, Resources, Methodology, Investigation, Formal analysis, Data curation, Conceptualization. **Haura Syabihah:** Visualization, Software, Resources, Methodology, Data curation. **Taufik Muhammad Fakih:** Writing – review & editing, Writing – original draft, Visualization, Validation, Supervision, Software, Resources, Methodology, Investigation, Formal analysis, Data curation, Conceptualization.

## Declaration of Competing Interest

The authors declare the following financial interests/personal relationships which may be considered as potential competing interests: Ratih Aryani reports was provided by Universitas Islam Bandung. Ratih Aryani reports a relationship with Universitas Islam Bandung that includes:. If there are other authors, they declare that they have no known competing financial interests or personal relationships that could have appeared to influence the work reported in this paper.

## Data Availability

The authors do not have permission to share data.
